# Integrated Radiologic, Pathologic, and Transcriptomic Analysis of Lymph Node Metastasis Potential in Small (≤3 cm) Lung Adenocarcinoma

**DOI:** 10.3390/cancers18162720

**Published:** 2026-08-21

**Authors:** Min Zhao, Yuan Li, Lingqi Gao, Wangjia Li, Hao Zhang, Dan Li, Fajin Lv, Shugeng Gao

**Affiliations:** 1 Department of Radiology, The First Affiliated Hospital of Chongqing Medical University, Chongqing 400016, China; zgcq13zm@163.com (M.Z.); shiyoululiyuan@163.com (Y.L.); gaolingqi2024@163.com (L.G.); 18883938636@163.com (W.L.); 2 Department of Cardiothoracic Surgery, The First Affiliated Hospital of Chongqing Medical University, Chongqing 400016, China; 3 Department of Thoracic Surgery, National Cancer Center/National Clinical Research Center for Cancer/Cancer Hospital, Chinese Academy of Medical Sciences and Peking Union Medical College, Beijing 100021, China; zhanghao@cicams.ac.cn; 4 Clinical Pathology Laboratory of Pathology Diagnostic Center, Chongqing Medical University, Chongqing 400016, China; 18375898495@163.com

**Keywords:** lung adenocarcinoma, multimodal, single-cell analysis, tumor microenvironment, lymph node metastasis

## Abstract

**Background**: This study aimed to develop and validate a multimodal radiology–pathology model (MoM-LNM) that integrates preoperative computed tomography (CT) radiomics and postoperative pathomics to identify lymph node metastasis (LNM) in lung adenocarcinoma (LUAD). **Methods**: Patients were enrolled from two medical centers (Center A: *n* = 483; Center B: *n* = 153). Data from Center A were divided into training (*n* = 339), validation (*n* = 72) and internal test (*n* = 72) sets, while Center B served as the external test set. Radiomics, pathomics, and MoM-LNM were developed using a multilayer perceptron. The optimal cutoff value for risk stratification was determined from the best-performing model. Bulk RNA sequencing and single-cell RNA sequencing were used to explore the underlying biological characteristics. **Results**: The MoM-LNM achieved AUCs of 0.879–0.944 across the validation and test sets and showed the best overall performance. The high-risk group had significantly worse 3-year overall survival (92.1% vs. 98.8%) and recurrence-free survival (RFS) (73.8% vs. 90.6%) than the low-risk group (both *p* < 0.001). Among patients without LNM, the high-risk group remained independently associated with worse RFS (hazard ratio = 2.387, 95% confidence interval: 1.350–4.219). Bulk RNA sequencing and single-cell RNA sequencing showed distinct immune characteristics in the high-risk group. **Conclusions**: The MoM-LNM provides postoperative risk stratification in LUAD patients. Notably, among patients without pathologically confirmed LNM, it identified a subgroup at elevated risk of adverse outcomes.

## Simple Summary

Lymph node metastasis is a key prognostic factor in lung adenocarcinoma, yet conventional pathology may miss micrometastasis, and some node-negative patients still experience recurrence. To address this, this study developed a multimodal model integrating CT radiomics and pathological images to evaluate the risk of lymph node metastasis in patients with small lung adenocarcinoma. The model stratified patients into high- and low-risk groups and, in exploratory analyses, the high-risk subgroup among node-negative patients showed significantly worse recurrence-free survival, suggesting that the model captures aggressive tumor biology beyond conventional nodal staging. Transcriptomic analyses further revealed distinct immune microenvironment features in high-risk tumors, providing biological support for the model. This approach may enhance postoperative risk stratification and inform surveillance strategies, particularly for node-negative patients at elevated risk.

## 1. Introduction

Lung adenocarcinoma (LUAD) is the predominant histopathological subtype of lung cancer [1], with substantial variability in prognostic outcomes among patients. Although some patients achieve a 5-year survival rate of 100% after surgery [2], overall survival (OS) in LUAD remains below 20% [3]. This heterogeneity is largely driven by tumor progression and metastasis, which adversely impact patients’ quality of life and survival [4,5]. Accurate detection of lymph node metastasis (LNM) is therefore important for treatment planning and risk assessment.

The incidence of LNM in lung cancer cases with lesions ≤ 2 cm in diameter has been reported to be about 12.3% [6]. However, accurate assessment of LNM has several limitations. First, some patients with invasive adenocarcinoma (IAC) cannot undergo lymph node dissection due to poor pulmonary function, and the extent of dissection also depends on the surgeon’s experience. Second, lymph node accessibility varies among individuals, and in some patients the nodes are difficult to identify, which makes complete dissection challenging. Finally, postoperative assessment of LNM relies on a single hematoxylin and eosin (HE) stained paraffin section, which provides only a static and localized snapshot of the tumor. Consequently, micrometastases may be missed, which are associated with worse OS (55% vs. 75%) and higher recurrence risk [7]. Evidence also shows that a proportion of patients without pathologically detected LNM still experience recurrence, suggesting that conventional pathological nodal assessment may not fully capture the underlying disease burden in all patients. Therefore, complementary approaches for postoperative risk stratification may help identify patients at increased risk of adverse outcomes and support more individualized clinical management.

Computed tomography (CT) and histopathological whole-slide images (WSIs) are important sources of information for risk stratification of tumors. They capture macro- and micro-scale morphological patterns associated with patient outcomes [8]. Although radiology and pathology offer complementary perspectives, conventional assessments rely heavily on expert interpretation and may miss subtle morphological features. Radiomics and pathomics have thus emerged as powerful tools for extracting subvisual features and have shown prognostic potential [9,10].

Previous studies [11,12,13] have several limitations: (1) they often rely on single-modal data (CT or pathology alone); (2) external validation is often lacking; (3) biological interpretability is limited; and (4) few studies focus specifically on LNM. To address these gaps, this study aimed to develop and validate a multimodal radiology–pathology framework for LNM assessment in IAC using CT and HE-stained WSIs. We comprehensively evaluate model performance, establish postoperative risk stratification, and explore the biological characteristics associated with the identified risk groups.

## 2. Materials and Methods

This study included patients from The First Affiliated Hospital of Chongqing Medical University (center A; ZZ2025-009-01) and The Cancer Hospital of Chinese Academy of Medical Sciences (center B; No. 22/492-3694), with approval from their respective ethics committees. Written informed consent was waived for the retrospective datasets and was obtained for the bulk RNA sequencing (bulk RNA-seq) and single-cell RNA sequencing (scRNA-seq) datasets.

### 2.1. Patient Inclusion and Digitization

**Figure 1:**
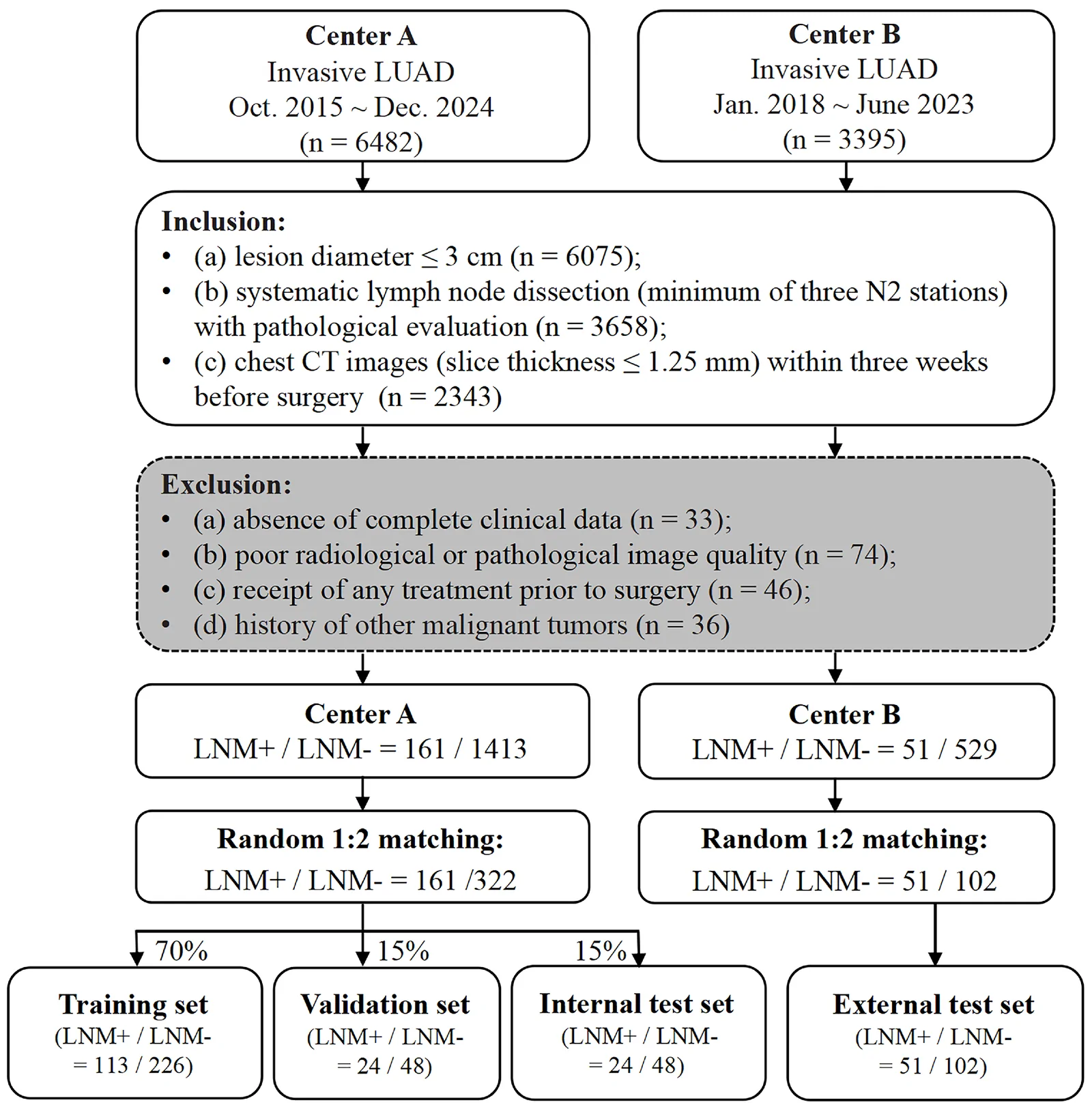
Flow chart of patient selection.

This retrospective study enrolled patients who underwent surgical resection and were pathologically diagnosed with IAC at two medical centers between October 2015 and December 2024. Inclusion criteria were: (a) lesion diameter ≤ 3 cm; (b) systematic lymph node dissection (minimum of three N2 stations) with pathological evaluation; and (c) chest CT images (slice thickness ≤ 1.25 mm) within three weeks before surgery. Exclusion criteria included: (a) absence of complete clinical data; (b) poor radiological or pathological image quality; (c) receipt of any treatment prior to surgery; and (d) history of other malignant tumors. The patient selection process is summarized in Figure 1. Pathologically confirmed patients with no lymph node metastasis (pN0) were randomly selected at a 1:2 ratio using the “pandas package (v 2.2.3)” with a fixed random seed to ensure reproducibility.

Eligible patients undergoing routine surgical resection for IAC at Center A were prospectively enrolled for fresh tissue collection between March 2025 and May 2025 after providing written informed consent. All transcriptomic specimens were obtained from surplus tumor tissue remaining after completion of clinically required pathological sampling. No additional tissue was collected beyond standard clinical practice, and the specimen did not interfere with surgery, pathological evaluation, or patient care. Cases were selected after pathological review to ensure balanced representation of high-risk and low-risk tumors. For exploratory scRNA-seq analysis, fresh tumor tissues from three high-risk and three low-risk patients were obtained from surplus surgical specimens. For exploratory bulk RNA-seq analysis, an independent cohort comprising sixteen fresh-frozen tumor samples (eight high-risk and eight low-risk cases) was collected during the same period.

Clinical information, preoperative CT images, and postoperative histopathological slides were systematically collected for all enrolled patients. Retrospectively collected patients from Center A between October 2015 and December 2024 were randomly divided into a training set (70%), validation set (15%) and internal test set (15%). Those from Center B between January 2018 and June 2023 constituted the external test set. The database was locked in May 2025 prior to dataset partitioning and model development.

### 2.2. Clinical Information

Clinical, radiological, and pathological data were collected, including age, sex, smoking, clinical T staging, pure solid nodules, lobulation, spiculation, air bronchogram sign, histological grade, tertiary lymphoid structures (TLS), lymphovascular invasion (LVI), spread through air spaces (STAS) and pleural invasion. Follow-up data were collected from electronic medical records and structured telephone interviews. The primary endpoints of this study were OS and recurrence-free survival (RFS). RFS was defined as the interval from surgical resection to the first occurrence of disease recurrence. Patients were enrolled between 2015 and 2024 and followed until 2026.

### 2.3. Radiomics Feature Extraction and Selection

CT images were obtained in DICOM format from institutional Picture Archiving and Communication Systems (PACS). To reduce variation related to CT scanners and scanning protocols, all CT images underwent preprocessing, including intensity normalization and resampling. Tumor segmentation was performed semi-automatically by two radiologists, each with 15 years of experience in chest CT, using 3D-Slicer software (v5.6.2) under lung window settings (width: 1400 HU; level: −500 HU) [14]. All region-of-interest (ROI) outlines were subsequently reviewed and confirmed by a senior radiologist with 30 years of expertise.

A total of 1316 radiomics features were extracted from each segmented tumor volume using the PyRadiomics package (v3.1.0) in Python [15]. To prevent data leakage, all data-dependent feature preprocessing and selection steps were performed exclusively within each cross-validation training fold. Features were Z-score normalized using parameters estimated from the training fold, and those demonstrating both intra- and inter-observer reproducibility with intraclass correlation coefficients (ICCs) greater than 0.75 were retained. Subsequently, the Mann–Whitney U test was applied to the training fold to identify features with significant differences (*p* < 0.05), and Spearman correlation analysis was used to exclude highly redundant features (r > 0.9). Finally, the radiomics features were selected via the Least Absolute Shrinkage and Selection Operator (LASSO) regression method using the training fold. The resulting normalization parameters, selected feature indices, and LASSO coefficients were then fixed and applied without refitting to the corresponding validation fold, which was subsequently used to generate out-of-fold (OOF) predictions.

### 2.4. Pathomics Feature Extraction and Selection

A total of 1033 tumor WSIs were digitized using a KFBIO scanner ((KF-PRO-040-HI; Konfoong Bioinformation Tech Co., Ltd., Ningbo, China)). Tumor regions were manually annotated on each WSI using QuPath software (v0.5.2). Given the high resolution of WSIs (ranging from 10,000 to 100,000 pixels per dimension), which precludes direct input into deep neural networks, the annotated tumor areas were divided into non-overlapping 512 × 512 pixel tiles at 400× magnification. Tissue-free tiles were excluded by applying Otsu’s thresholding method [16]. To reduce staining variation and batch effects, all tiles underwent color normalization via Vahadane’s method [17] with a predefined reference image that was fixed before model development. Before any data-dependent preprocessing, representation learning, or model training, patients were assigned to their respective dataset partitions and cross-validation folds.

A pretrained ResNet50 convolutional neural network was employed to extract a 2048-dimensional feature vector from each tile, with its final fully connected layer removed. An attention-based multiple-instance learning module was then used to aggregate the tile-level features into a 512-dimensional WSI-level representation. For patients with multiple WSIs, the resulting WSI-level pathomics feature vectors were aggregated using mean pooling to generate a single patient-level representation.

To prevent information leakage during dimensionality reduction, all data-dependent representation-learning and preprocessing steps were performed exclusively within the corresponding cross-validation training fold. Z-score normalization parameters were likewise estimated exclusively from the training fold and applied unchanged to the validation fold. The autoencoder [18] was trained using only the patient-level representations in the training fold, and the learned parameters were subsequently fixed and applied to the corresponding validation fold without refitting. This procedure ensured that no information from the validation fold was used during representation learning or feature preprocessing.

### 2.5. Model Construction

All preprocessing, feature selection, dimensionality reduction, representation learning, and other trainable steps were strictly performed within each cross-validation training fold. The trained pipelines were applied to the corresponding validation folds to generate OOF predictions, which served directly as the radiomics and pathomics signatures for the meta-model. The complete pipeline is shown in Supplementary Figure S1.

This study constructed radiomics, pathomics and multimodal radiology–pathology model (MoM-LNM) by multilayer perceptron (MLP) method [19]. The multimodal MoM-LNM model was constructed using an OOF stacking strategy rather than feature-level fusion. Specifically, the OOF predictions generated by the radiomics and pathomics base models served as the radiomics and pathomics signatures, respectively. These OOF signatures were the sole inputs to the MoM-LNM meta-model, ensuring that the meta-model did not directly access the original radiomics or pathomics feature spaces. Within each cross-validation training fold, hyperparameters were optimized using grid search with an inner validation procedure, and the selected hyperparameters were then fixed and applied to the corresponding outer validation fold. All three models adopted an MLP architecture consisting of two hidden layers (64 and 32 neurons, respectively) with ReLU activation, culminating in a sigmoid output layer for binary classification. The optimized hyperparameters and final architectures for the radiomics, pathomics, and multimodal models are provided separately in Appendix A.

### 2.6. Model Performance Evaluation

Model discrimination was evaluated using receiver operating characteristic (ROC) curve analysis. Sensitivity, specificity, positive predictive value (PPV), and negative predictive value (NPV) were reported as key metrics. The 95% confidence intervals (CIs) for the areas under the curves (AUCs) were estimated using bootstrap resampling with 1000 iterations. Briefly, 10-fold cross-validation was performed within the training set to generate OOF predictions from the base learners, and these predictions were subsequently used as inputs for training the meta-learner. Model performance was evaluated using ROC analysis, and the robustness of the model was further assessed based on the performance across the 10 folds. DeLong’s test method was used to compare models’ performance. Clinical utility was assessed using decision curve analysis (DCA), which estimates the net benefit across a range of threshold probabilities. Finally, model interpretability was assessed using SHapley Additive exPlanations (SHAP) [20] analysis with the KernelExplainer. The final layer of the multilayer perceptron generated a logit output for the pathologically confirmed nodal metastasis (pN+) class, which was transformed into predicted probabilities using the sigmoid function. SHAP values were calculated using the predicted probability output with a logit link function and were therefore represented on the log-odds scale. Positive and negative SHAP values indicated increased and decreased contributions to the predicted risk of LNM, respectively. The background dataset consisted of a randomly selected subset of up to 200 patients from the training set (random seed = 42), processed using the same preprocessing pipeline and feature representation as the explained samples. The best-performing model was selected for LNM risk assessment in early-stage LUAD.

The calibration of the optimal model was assessed by evaluating the agreement between predicted probabilities and observed LNM outcomes. Calibration performance was quantified using the Brier score, calibration intercept, and calibration slope. The calibration intercept and slope were estimated by fitting a logistic regression model with the observed LNM status as the dependent variable and the logit-transformed predicted probabilities as the independent variable. Calibration curves were generated by plotting the observed event probabilities against the predicted probabilities using locally estimated scatterplot smoothing. For the training set, calibration metrics were calculated using out-of-fold predictions to reduce optimism due to model overfitting. Calibration performance was further evaluated in the independent validation, internal test, and external test set.

### 2.7. Risk Stratification and Group Comparison

Based on the optimal model, a novel risk stratification, MoM-LNM was conducted. The optimal cutoff value was determined by maximizing the Youden index from the training set’s ROC curve, and all patients were subsequently categorized into high- or low-risk groups. The prognostic significance of the risk group for OS and RFS was evaluated using Kaplan–Meier analysis and log-rank tests. Furthermore, to investigate the biological mechanisms underlying the model-derived risk stratification, we performed exploratory analysis involving radiological, pathological, immune, and genomic characteristics. These analyses were performed independently for biological exploration and were not used in model development.

### 2.8. Single-Cell RNA Sequencing

ScRNA sequencing was performed to explore potential differences in cellular composition and biological states between the two risk groups. 3 high-risk and 3 low-risk tumor samples were collected from Center A (between March 2025 and May 2025) specifically for scRNA-seq (Supplementary Table S1). Detailed information on sequencing procedures is provided in Appendix B.

Seurat was used for quality control, normalization, and clustering of tumor cells, with gene expression normalized using the “LogNormalize” method, followed by principal component analysis, construction of a shared nearest neighbor graph, Louvain clustering, and uniform manifold approximation and projection (UMAP) visualization. Differentially expressed genes (DEGs) among tumor cell clusters were identified using the Wilcoxon rank-sum test implemented in Seurat, comparing all cells from high-risk samples versus all cells from low-risk samples. Given the limited cohort size (*n* = 3 per group), a pseudobulk analysis (aggregating counts per patient) was not performed, as it would lack sufficient degrees of freedom for reliable variance estimation and statistical inference. Cell–cell communication analysis was performed using the CellChat package (v1.6.1) with the CellChatDB human ligand–receptor interaction database. Normalized expression matrices were used to infer potential ligand–receptor interactions among major malignant and immune cell populations. Statistical significance was determined using the built-in cell-level permutation framework implemented in CellChat, with *p* values adjusted using the Benjamini–Hochberg method. The resulting *p* values reflect cell-level permutation testing rather than comparisons across independent patients. Interactions with adjusted *p* values < 0.05 were considered significant. Sender and receiver cell populations were defined according to ligand and receptor expression patterns, respectively, and significant interactions were visualized using bubble plots. Tumor cell differentiation status was assessed using CytoTRACE(v1.1.0), and differentiation states were evaluated and visualized across tumor cell clusters and risk groups. Cell-type composition was summarized descriptively based on the proportion of annotated cells within each risk group. No formal statistical comparisons of cell-type proportions were performed because the scRNA-seq cohort comprised only six independent biological samples (three patients per group).

### 2.9. Bulk RNA-Seq

Bulk RNA-seq was utilized to explore tumor heterogeneity between the two groups. A separate cohort of 8 high-risk and 8 low-risk fresh-frozen IAC tumor samples was collected from Center A (between March 2025 and May 2025) specifically for Bulk RNA-seq (Supplementary Table S2). Detailed information on sequencing procedures is provided in Appendix C.

To identify molecular alterations underlying the risk stratification, differential gene expression analysis was performed between high- and low-risk tumors using the ‘limma’ R package (v3.66.0) with voom normalization to account for heteroscedasticity in count data. Genes with an adjusted *p*-value (FDR) < 0.1 and absolute log2 fold change (|log2FC|) > 1.5 were considered DEGs. Functional interpretation of the DEGs was carried out through GO and KEGG pathway enrichment analysis. Additionally, Gene Set Enrichment Analysis (GSEA) was performed using hallmark gene sets from the Molecular Signatures Database (‘clusterProfiler’ R package(v4.19.8)). Additionally, the single-sample GSEA (ssGSEA) method was used to quantify 28 types of infiltrating immune cells using transcriptomics data and associated gene sets (‘GSVA’ R package (v2.4.4)).

### 2.10. Statistical Analysis

Statistical analyses were performed using Python (v3.9.1) and R (v4.4.1). Continuous variables following a normal distribution are expressed as mean ± standard deviation (SD) and compared using Student’s *t*-test. Non-normally distributed variables are presented as median (interquartile range) and compared using the Mann–Whitney U test. Categorical variables are presented as frequencies and percentages, and compared using the Chi-square test or Fisher’s exact test, as appropriate. The correlation between MoM-LNM and established features using Spearman correlation and binary logistic regression analysis. The correlation effects were measured by correlation coefficient (rs) and odds ratio (OR). Univariable and multivariable Cox regression analyses were performed to evaluate the associations of the MoM-LNM risk group and clinicopathological factors with OS and RFS. The proportional hazards assumption was tested using Schoenfeld residuals. Standard Cox models were applied when the assumption held; otherwise, an extended Cox model with a log(time)–interaction term was used for the primary exposure (MoM-LNM risk group), and non-proportional covariates were adjusted via stratification. Time-specific hazard ratios (HRs) with 95% confidence intervals (CIs) for the MoM-LNM risk group at 12, 24, and 36 months were reported when the extended model was applied. Survival differences between groups were assessed using Kaplan–Meier survival analysis and the log-rank test. Univariable and multivariable Cox proportional hazards regression analyses were performed to identify independent prognostic factors associated with OS and RFS. HRs with 95% CIs were calculated. A *p*-value < 0.05 was considered statistically significant.

To address potential confounding due to imbalanced baseline clinicopathological characteristics between the high-risk and low-risk groups, propensity score matching (PSM) was performed as a prespecified sensitivity analysis. Propensity scores were estimated using a logistic regression model with the MoM-LNM risk group (high-risk vs. low-risk) as the dependent variable and the following covariates: tumor size, LVI, STAS, pleural invasion, tumor differentiation, and adjuvant therapy. One-to-one nearest neighbor matching was performed without replacement, with a caliper width of 0.2 standard deviations of the logit of the propensity score. Covariate balance before and after matching was assessed using standardized mean differences (SMDs), with an SMD < 0.1 indicating adequate balance. Kaplan–Meier survival analysis and Cox proportional hazards regression were subsequently repeated in the matched cohort. Additional sensitivity analysis was conducted using alternative caliper widths (0.1) and matching ratios (1:2) to evaluate the robustness of the findings.

## 3. Results

### 3.1. Study Population and Characteristics

A total of 636 patients were enrolled. Disease recurrence was observed in 22.6% (144/636) of patients, of whom 47.9% (69/144) did not have LNM. Among all patients, 82.2% had three-year follow-up data. Baseline clinicopathological characteristics are summarized in Table 1.

### 3.2. Feature Selection and Model Construction

After dimensionality reduction, 6 radiomics features and 64 pathomics features remained (Supplementary Figure S2). Grid search on the training set identified the optimal hyperparameters for the radiomics and pathomics models. Using the selected features and optimized hyperparameters, the radiomics, pathomics, and radiology–pathology models were subsequently developed.

### 3.3. Performance Evaluation of All Models

**Figure 2:**
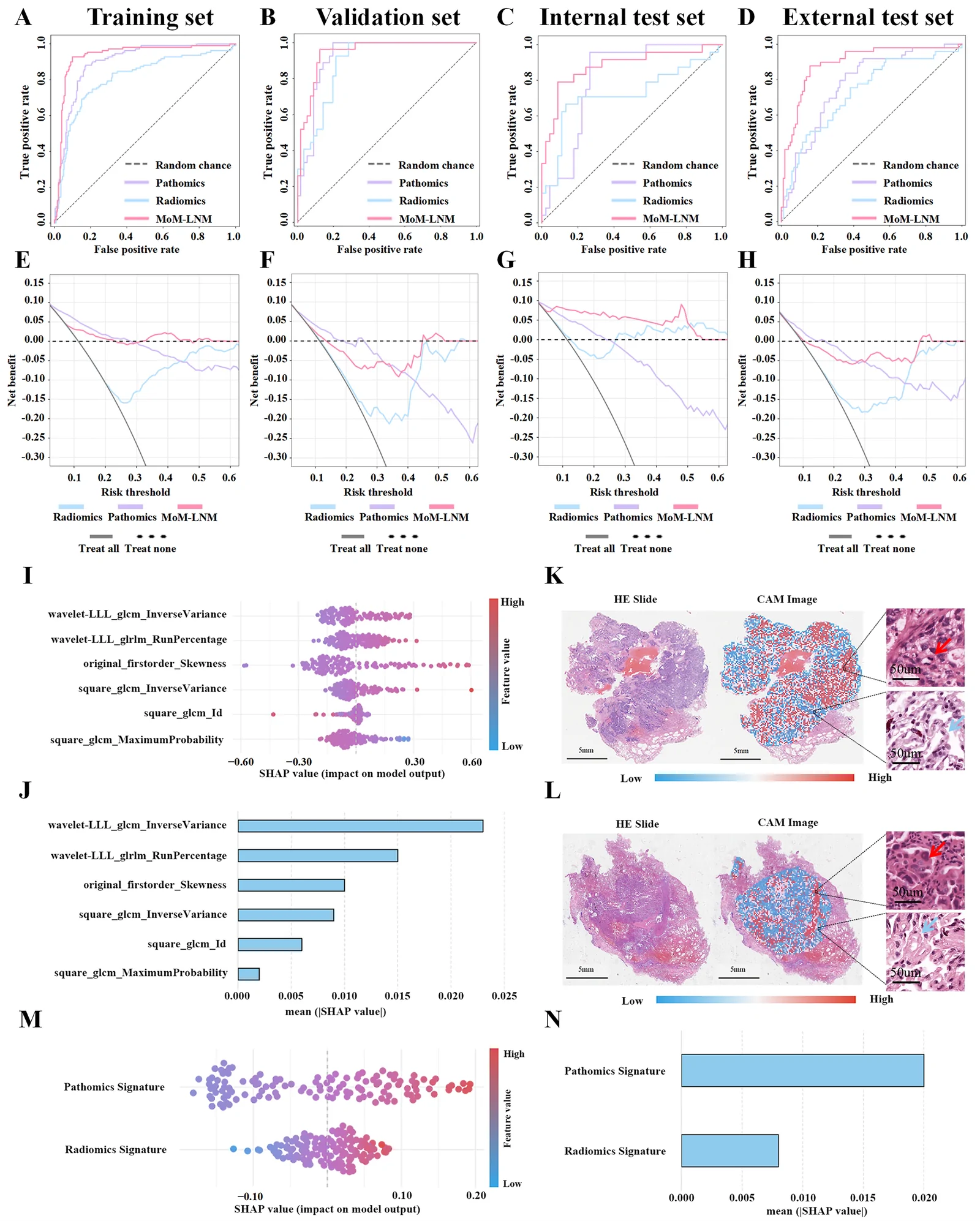
ROC, DCA curves and visualization for models. (**A**–**D**) ROC curves of the radiomics, pathomics, and multimodal radiology–pathology models for assessing LNM in the training set (**A**), validation set (**B**), internal test set (**C**), and external test set (**D**). (**E**–**H**) DCA of the three models in the training set (**E**), validation set (**F**), internal test set (**G**), and external test set (**H**), calculated at the true population prevalences of 10.2% (Center A) and 8.8% (Center B). (**I**,**J**) The SHAP bar of radiomics features; (**K**,**L**) Representative CAM visualizations. The red-highlighted high-attention regions predominantly corresponded to aggressive histological growth patterns, including micropapillary (red arrow in (**K**)) and solid (red arrow in (**L**)) patterns, whereas the low-attention regions were primarily associated with lepidic growth patterns (blue arrow in (**K**,**L**)) (Hematoxylin and Eosin stain, 1× or 400×); (**M**,**N**) SHAP summary plot of the radiomics signature and pathomics signature. ROC, receiver operating characteristic; DCA, decision curve analysis; LNM, lymph node metastasis; SHAP, Shapley additive explanations; CAM, class activation mapping.

The radiomics model for assessing LNM showed AUC of 0.808 (95% CI, 0.753–0.856; SD: 0.058) in the training set, 0.891 (95% CI: 0.821–0.950) in the validation set, 0.722 (95% CI: 0.567–0.855) in the internal test set and 0.730 (95% CI: 0.630–0.821) in the external test set (Figure 2A–D). SHAP analysis demonstrated that the wavelet-LLL glcm InverseVariance was the most important radiomics feature and had a positive contribution to model predictions (Figure 2I,J).

The pathomics model for LNM identification achieving AUCs of 0.897 (95% CI: 0.859–0.930; SD: 0.041) in the training set, 0.921 (95% CI: 0.861–0.973) in the validation set, 0.807 (95% CI: 0.698–0.896) in the internal test set, and 0.776 (95% CI: 0.691–0.855) in the external test set (Figure 2A–D). Figure 2K,L demonstrates the class activation mapping image showing the areas that the model determined as significant for LNM.

The MoM-LNM yielded AUC values of 0.933 (95% CI: 0.899–0.961; SD: 0.053) in the training set, 0.944 (95% CI: 0.894–0.986) in the validation set, 0.879 (95% CI: 0.772–0.959) in the internal test set, and 0.893 (95% CI: 0.831–0.947) in the external test set (Figure 2A–D). SHAP demonstrated that the pathomics model contributed more substantially to the MoM-LNM than the radiomics model (Figure 2M,N).

Among the three models, the MoM-LNM shows superior AUC values, accuracy, sensitivity, specificity, PPV and NPV (Supplementary Table S3). Notably, the PPV and NPV were adjusted to reflect the source-population prevalence (10.2% for Center A; 8.8% for Center B), as presented alongside the original matched-cohort metrics. Supplementary Table S4 provides the confusion matrix of the MoM-LNM. DeLong’s test showed that the MoM-LNM significantly outperformed the radiomics model in the training, internal test, and external test cohorts (all *p* < 0.05; Supplementary Table S5). Compared with the pathomics model, a statistically significant improvement was observed only in the external test cohort (*p* = 0.034), although the MoM-LNM consistently achieved higher AUCs across all cohorts. DCA, recalculated at these true prevalences, demonstrated a modest positive net benefit within a limited range of low risk thresholds, which was superior to the radiological or pathological models alone (Figure 2E–H). DCA curves before prevalence adjustment are shown in Supplementary Figure S3.

Calibration metrics before and after prevalence adjustment are summarized in Supplementary Table S6. Calibration curves before and after prevalence adjustment are summarized in Supplementary Figure S4. Recalibration to real-world prevalence (10.2% internal; 8.8% external) yielded minimal changes in the external set: the Brier score shifted from 0.167 to 0.166 and the intercept from 1.222 to 1.215. The calibration slope remained unchanged at 0.751 (95% CI 0.499–1.002).

### 3.4. Risk Stratification and Subgroup Analysis

**Figure 3:**
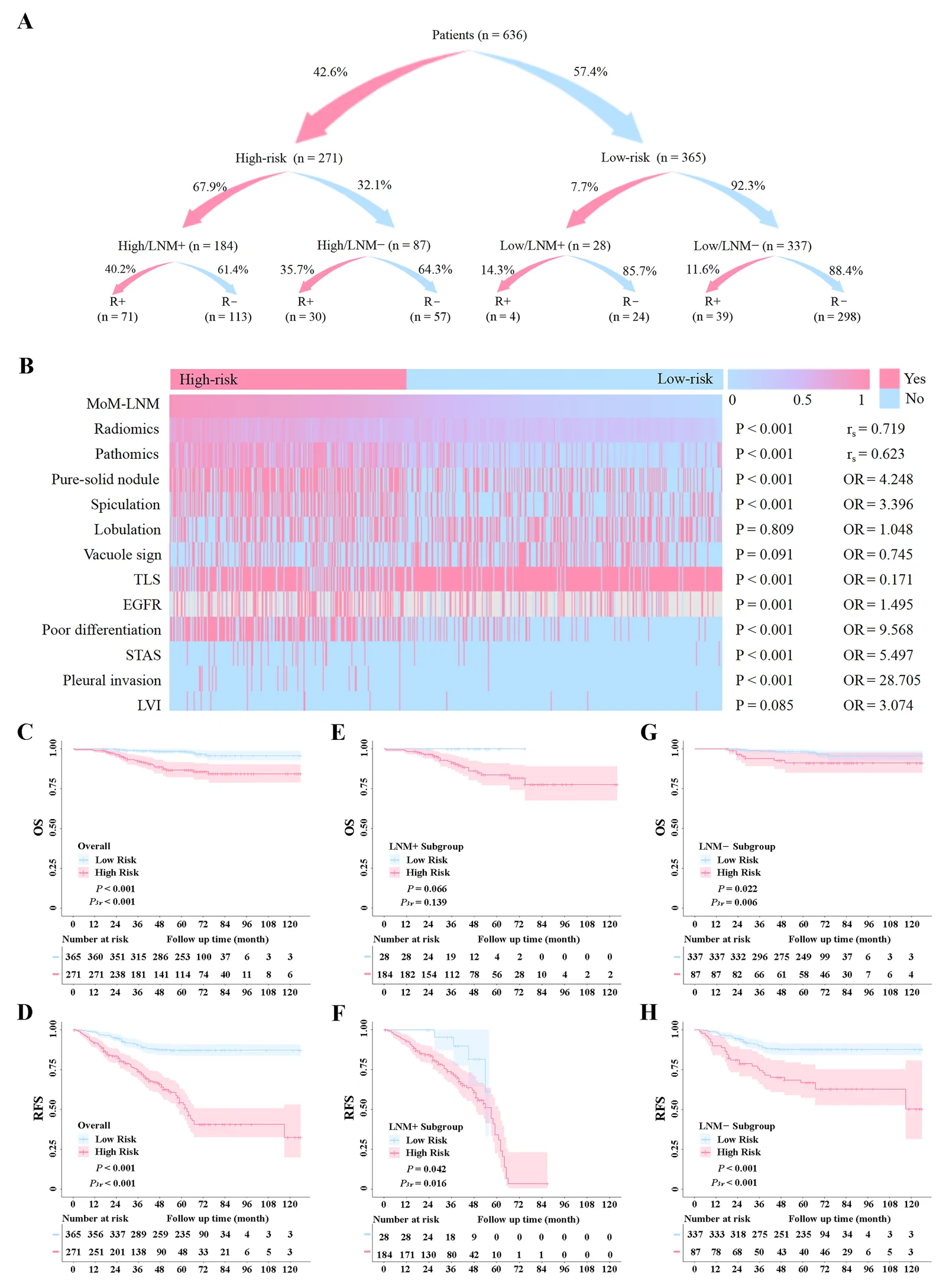
Subgroup analysis based on multimodal model risk stratification. (**A**) Patient subgroups by model-identified LNM risk, LNM status, and recurrence status. (**B**) Correlation heatmap of radiological/pathological features with model‑assigned risk. (**C**–**H)** Kaplan‑Meier curves for OS and RFS comparing risk groups in the overall cohort (**C**,**D**), LNM+ (**E**,**F**), and LNM− (**G**,**H**) subgroups. For panels (**C**–**H**), both overall log‑rank *p* and 3‑year milestone *p* (*p*_3_*_Y_*) are reported. TLS, tertiary lymphoid structures; STAS, spread through air spaces; LVI, lymphovascular invasion; OS, overall survival; RFS, recurrence-free survival; LNM, lymph node metastasis; R, recurrence.

MoM-LNM was selected as the optimal model and used to stratify patients into high-risk (*n* = 271) and low-risk (*n* = 365) groups based on the optimal cutoff value (0.352) (Figure 3A). This study further elucidated the underlying mechanisms of risk stratification through visualization and correlation analysis with various radiological and pathological characteristics relevant to LNM. Feature maps showed the distribution of these characteristics across the two risk groups (Figure 3B). The high-risk group was more frequently associated with pure-solid nodules, spiculation sign, poor differentiation, STAS, and pleural invasion. The high-risk group also had significantly worse long-term outcomes, with lower 3-year OS (92.1% vs. 98.8%, *p* < 0.001; Figure 3C) and 3-year RFS (73.8% vs. 90.6%, *p* < 0.001; Figure 3D) than the low-risk group. In addition, recurrence occurred in 37.3% (101/271) of patients in the high-risk group but only 11.8% (43/365) of the low-risk group. To further assess the robustness and generalizability of the MoM-LNM model, survival analysis was independently conducted in the external validation set (*n* = 153). The model consistently stratified patients into distinct prognostic groups, with the high-risk group showing significantly poorer OS and RFS than the low-risk group (Supplementary Figure S5A,B). These findings demonstrate that the prognostic performance of the MoM-LNM model is reproducible in an independent external test set.

Patients were further categorized into four subgroups according to LNM status: high-risk with LNM (High/LNM+), high-risk without LNM (High/LNM−), low-risk with LNM (Low/LNM+), and low-risk without LNM (Low/LNM−) (Figure 3A). The High/LNM+ group showed numerically lower 3-year OS (91.2% vs. 100.0%, *p* = 0.139; Figure 3E) and RFS (73.0% vs. 95.5%, *p* = 0.016; Figure 3F) than the Low/LNM+ group. Importantly, the High/LNM− group had significantly lower 3-year OS (93.9% vs. 98.7%, *p* = 0.006 Figure 3G) and RFS (73.5% vs. 90.2%, *p* < 0.001; Figure 3H) than the Low/LNM− group.

Finally, patients were further divided into eight subgroups based on recurrence status: High/LNM+/R+, High/LNM+/R−, High/LNM−/R+, High/LNM−/R−, Low/LNM+/R+, Low/LNM+/R−, Low/LNM−/R+, and Low/LNM−/R− (Figure 3A). The High/LNM−/R− group had a significantly shorter median follow-up duration than the High/LNM−/R+ group (47.5 vs. 66 months, *p* = 0.017).This suggests that the absence of observed recurrence in this subgroup may be attributable to limited follow-up duration rather than a genuinely lower risk. Extended follow-up in future studies will be valuable to clarify this finding.

Univariable and multivariable Cox regression analyses were performed to evaluate the prognostic value of the MoM-LNM signature (Table 2). The distribution of adjuvant therapy across the MoM-LNM risk groups is shown in Supplementary Table S7. During follow-up, a total of 38 deaths (OS events) occurred in the entire cohort, including 29 in the high-risk group and 9 in the low-risk group. Additionally, 144 recurrences (RFS events) were observed, with 101 in the high-risk group and 43 in the low-risk group. The proportional hazards assumption held for OS (global *p* = 0.830) but was violated for RFS (global *p* = 1.400 × 10^−8^), specifically for the MoM-LNM risk group, LNM, and poor differentiation. Consequently, an extended Cox model incorporating a log(time)-interaction term for the risk group and stratifying by LNM and poor differentiation was used for RFS. In this model, the high-risk group exhibited significantly elevated recurrence risks at 12 months (HR = 3.234, 95% CI: 1.669–6.267, *p* < 0.001), 24 months (HR = 2.510, 95% CI: 1.536–4.100, *p* < 0.001), and 36 months (HR = 2.156, 95% CI: 1.195–3.887, *p* = 0.011), with effects attenuating thereafter. The MoM-LNM risk group was also independently associated with OS after multivariable adjustment (HR = 5.128, 95% CI: 1.859–14.286, *p* = 0.002).

An additional Cox regression analysis was performed in all pN0 patients across the entire study cohort. The proportional hazards assumption was assessed using Schoenfeld residuals, and no significant time-dependent effect of the MoM-LNM risk group was observed in this subgroup. In the multivariable model, the MoM-LNM high-risk group remained independently associated with worse RFS (HR = 2.387, 95% CI: 1.350–4.219, *p* = 0.003), together with poorly differentiation (HR = 1.971, 95% CI: 1.134–3.425, *p* = 0.016), pleural invasion (HR = 11.486, 95% CI: 2.531–52.133, *p* = 0.002), STAS (HR = 4.217, 95% CI: 1.234–14.415, *p* = 0.022), and LVI (HR = 6.776, 95% CI: 1.531–29.979, *p* = 0.012). No independent association between the MoM-LNM risk group and OS was observed, although this analysis should be interpreted as exploratory because of the limited number of OS events.

A conventional clinicopathological model was constructed using six routinely available variables: tumor diameter, differentiation, LNM, LVI, STAS, and pleural invasion. The prognostic performance of this model was then compared with that of a corresponding model additionally incorporating MoM-LNM. For OS, the C-index increased from 0.702 to 0.754 (ΔC-index, 0.052), although the difference in C-index did not reach statistical significance (*p* = 0.188). Nevertheless, the likelihood ratio test demonstrated a significant improvement in model fit after adding MoM-LNM (χ^2^ = 6.146, *p* = 0.013). For RFS, the C-index increased from 0.673 to 0.694 (ΔC-index, 0.021; 95% CI, 0.005–0.060; *p* = 0.006), with a significant improvement in model fit (χ^2^ = 22.655, *p* < 0.001). Detailed comparisons of model performance are presented in Supplementary Table S8.

After 1:1 nearest neighbor PSM with a caliper of 0.200, 155 high-risk patients were successfully matched to 155 low-risk patients, yielding a matched cohort of 310 patients. All matching covariates achieved adequate balance after matching, with SMDs < 0.100 for LVI, STAS, pleural invasion, differentiation, adjuvant therapy and tumor size. In the matched cohort, Kaplan–Meier analysis demonstrated that the high-risk group had significantly worse OS (93.1% vs. 99.2% *p* < 0.001; Supplementary Figure S5C) and RFS (78.5% vs. 89.3% *p* < 0.001; Supplementary Figure S5D) compared with the low-risk group. Univariable Cox regression analysis confirmed that high-risk status was associated with a significantly increased risk of death (HR = 6.957, 95% CI: 2.037–23.758, *p* = 0.002) and recurrence (HR = 3.539, 95% CI: 2.071–6.047, *p* < 0.001). Multivariable Cox regression adjusting for all matching covariates in the matched cohort yielded consistent results for OS (HR = 7.162, 95% CI: 2.093–24.511, *p* = 0.002) and RFS (HR = 3.504, 95% CI: 2.040–6.019, *p* < 0.001). To verify the robustness of PSM findings, sensitivity analysis was performed using alternative matching parameters. A narrower caliper of 0.100 was applied (*n* = 294), the association remained significant for both OS (HR = 6.393, 95% CI: 1.848–22.112, *p* = 0.003) and RFS (HR = 3.636, 95% CI: 2.034–6.498, *p* < 0.001). Similarly, 1:2 matching (*n* = 379) yielded consistent results for OS (HR = 5.999, 95% CI: 2.211–16.275, *p* < 0.001) and RFS (HR = 3.581, 95% CI: 2.225–5.762, *p* < 0.001). These consistent findings across different matching strategies support the robustness of the association between MoM-LNM risk stratification and survival outcomes.

### 3.5. Exploration of Biological Mechanisms

**Figure 4:**
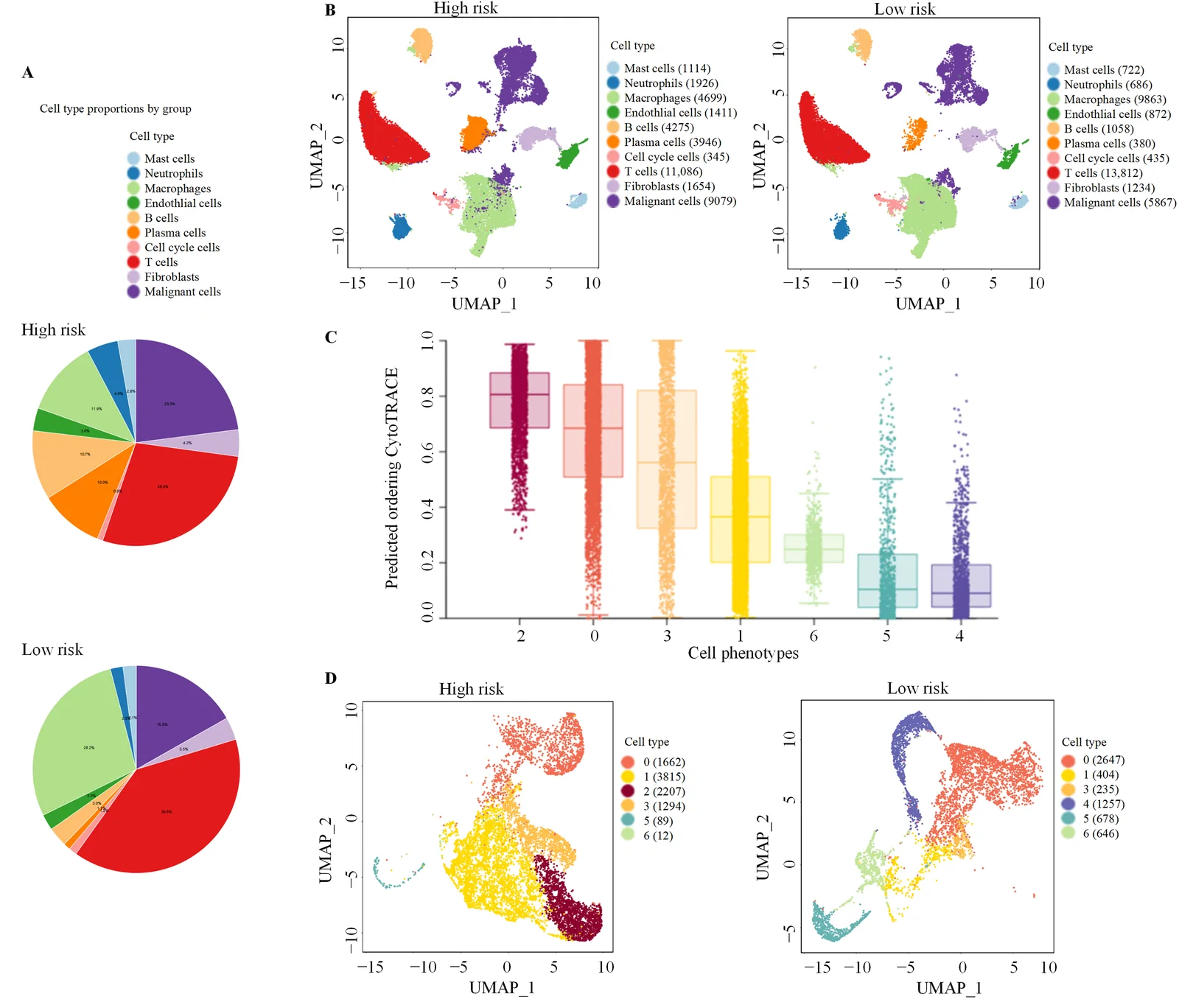
Single-cell analysis of high and low-risk groups. (**A**,**B**) Proportions of ten identified cell types presented across different groups. (**C**,**D**) Differentiation of malignant cells. UMAP, uniform manifold approximation and projection.

scRNA-seq analysis identified 74,464 single cells, which were annotated as malignant cells, B cells, plasma cells, neutrophils, endothelial cells, fibroblasts, mast cells, macrophages, cell cycle cells, and T cells. Compared with the low-risk group, the high-risk group exhibited higher proportions of malignant cells (23% vs. 16.8%), B cells (10.7% vs. 3%), and plasma cells (10% vs. 1.0%), along with markedly decreased proportions of macrophages (11.9% vs. 28.2%) and T cells (28% vs. 39.6%) (Figure 4A,B). The distribution of cells from 6 patients is shown in Supplementary Figure S6.

Tumor cells were classified into seven clusters and ordered along a differentiation trajectory using CytoTRACE, from poorly differentiated to highly differentiated states (Figure 4C). The high-risk group had a higher proportion of poorly differentiated tumor cells. The least differentiated subtype (C2) was found only in the high-risk group and showed significantly higher copy number variations (Figure 4D). In contrast, the low-risk group was enriched with well-differentiated tumor cells. Low-dimensional visualization also showed a continuous differentiation gradient across clusters (Figure 4D). The major enriched pathways of these seven subtypes are shown in Supplementary Figure S7.

Comparative analysis of ligand–receptor communication patterns between the high- and low-risk groups suggested that malignant cells exhibited stronger computationally inferred communication signals with macrophages, B cells, and T cells in the high-risk group. Several observed interactions, including MIF-CD74/CXCR4, LAMC1-CD44, and MIF-CD74/CD44, were enriched in the high-risk group (Supplementary Figure S8). These interactions have been previously implicated in tumor progression and immune regulation [21,22]; however, their functional roles require further experimental validation.

**Figure 5:**
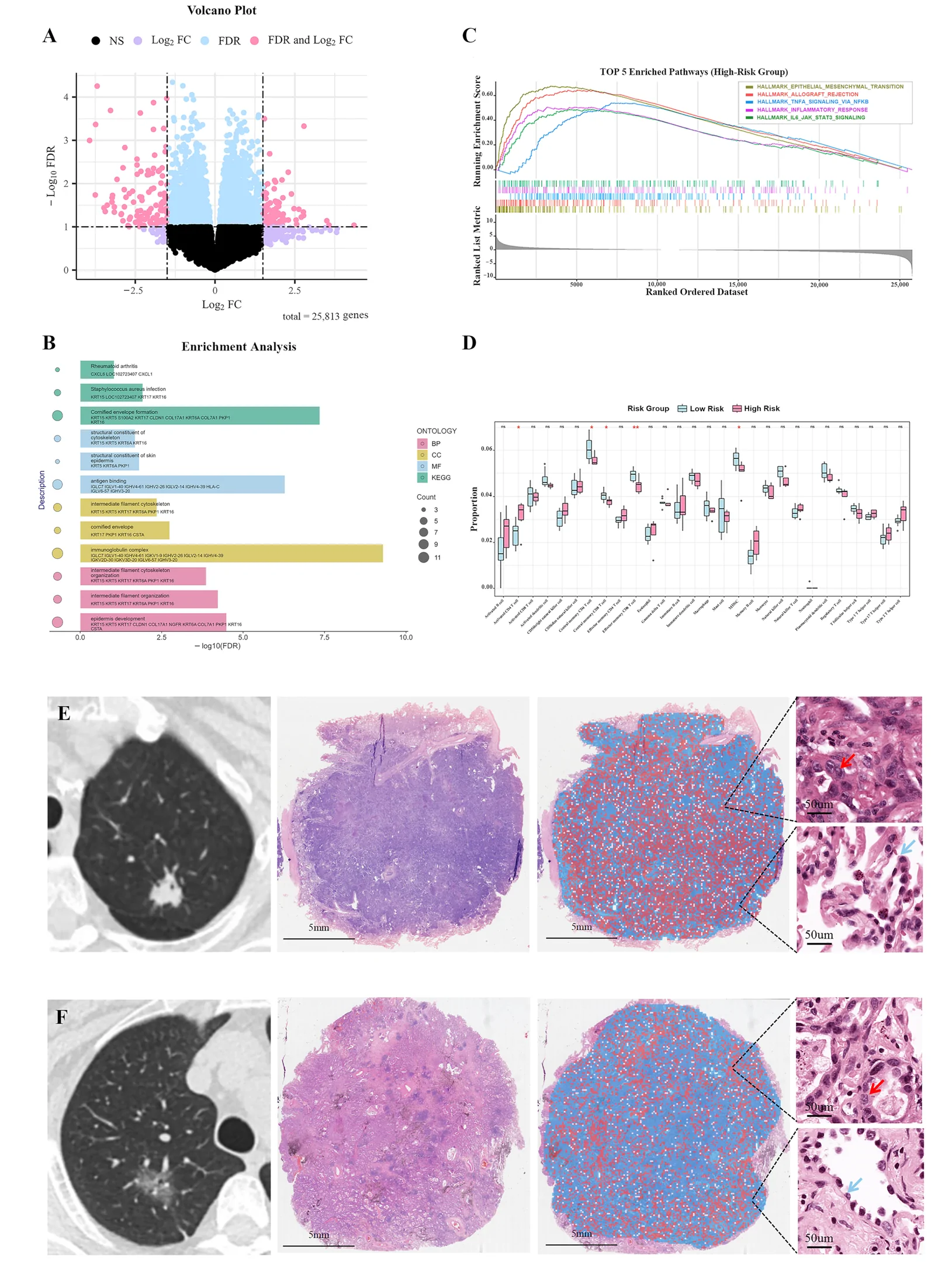
Biological interpretation of the multimodal radiology–pathology model. (**A**) Volcano plot showing DEGs between low- and high-risk groups. (**B**) Gene Ontology and (**C**) KEGG pathway enrichment analysis of DEGs. (**D**) The results of GSEA and ssGSEA between risk groups. * *p* < 0.05, ** *p* < 0.01. (**E**) Representative high-risk patient with LNM presenting as a solid nodule on CT. The high-attention regions predominantly corresponded to solid growth patterns (red arrow), whereas the low-attention regions were mainly associated with lepidic growth patterns (blue arrow) (Hematoxylin and Eosin stain, 1× or 400×). (**F**) Representative low-risk patient without LNM presenting as a ground-glass nodule on CT. The high-attention regions predominantly corresponded to acinar growth patterns (red arrow), whereas the low-attention regions were mainly associated with lepidic growth patterns (blue arrow) (Hematoxylin and Eosin stain, 1× or 400×). GSEA, Gene Set Enrichment Analysis; DEGs, differentially expressed genes; IAC, invasive adenocarcinoma; LNM, lymph node metastasis; CT, computed tomography.

A total of 463 DEGs were identified in the Bulk RNA-seq set between low- or high-risk groups (Figure 5A). Functional enrichment analysis demonstrated that these DEGs were mainly associated with epidermis development, intermediate filament organization, immunoglobulin complex, antigen binding, cornified envelope formation, Staphylococcus aureus infection, and rheumatoid arthritis (Figure 5B). GSEA further revealed that epithelial–mesenchymal transition, inflammatory response, TNFα signaling via NF-κB, IL6/JAK/STAT3 signaling, and allograft rejection were significantly enriched in the high-risk group (Figure 5C).

GO enrichment analysis showed significant enrichment of epidermis development, intermediate filament organization, immunoglobulin complex, antigen binding, and cornified envelope-related processes (Figure 5B). KEGG pathway analysis showed enrichment of cornified envelope formation, Staphylococcus aureus infection, and rheumatoid arthritis (Figure 5B). GSEA showed significant enrichment of epithelial–mesenchymal transition, inflammatory response, TNFα signaling via NF-κB, IL6/JAK/STAT3 signaling, and allograft rejection in the high-risk group (Figure 5C). Furthermore, immune infiltration analysis showed distinct immune cell compositions between two risk groups (Figure 5D). Figure 5E,F present radiographic and pathological images of a high-risk and a low-risk case, respectively, with different inflammatory cell counts.

## 4. Discussion

Accurate identification of LNM remains a critical challenge in the management of early-stage LUAD. In this study, we developed and validated MoM-LNM by integrating preoperative CT radiomics and postoperative pathomics to identify LNM and stratify patient risk. The combination of macro- and micro-scale morphological information improved performance over either modality alone, provided robust prognostic stratification, and identified distinct immune microenvironments between risk groups. Given that the model’s net benefit was positive only across specific risk-threshold ranges and that its pathomics component requires postoperative whole-slide images, MoM-LNM is primarily positioned as a postoperative risk-stratification tool, particularly for pN0 patients, rather than a preoperative tool for ruling out LNM or guiding biopsy.

Although pathological lymph node evaluation remains the clinical reference standard, pathology-negative status does not completely exclude occult metastatic potential. This limitation provides the rationale for developing complementary risk stratification tools. MoM-LNM may help identify a subset of pN0 patients with aggressive biological characteristics who require closer surveillance or further evaluation. A major strength of MoM-LNM is its multimodal design. Previous studies have often relied on radiomics alone [23,24], whereas our results suggest that adding pathomics improves predictive accuracy. MoM-LNM achieved high AUCs across internal (0.879) and external (0.893) test sets, supporting its generalizability. This improvement may reflect the complementary information captured by the two modalities. Radiomics characterizes the macroscopic tumor phenotype on CT, whereas pathomics captures microscopic architectural and cellular features from WSIs. The attention mechanisms in the pathomics pipeline also highlighted regions with high-grade growth patterns that are known to be associated with aggressive behavior [25,26]. These findings provide biological plausibility for the model predictions.

Beyond accurate LNM identification, the MoM-LNM was significantly associated with long-term survival and may have clinical implications for postoperative surveillance. By integrating imaging and pathological information, MoM-LNM may identify a distinct subgroup of pN0 patients with potential metastatic propensity and high recurrence risk, possibly representing an early high-risk state in the metastatic and recurrent process. This subgroup may provide a valuable entry point for investigating the molecular mechanisms and biological evolution of lung cancer recurrence and metastasis. Whether these high-risk pN0 patients may benefit from adjuvant therapy remains to be prospectively evaluated. This finding further suggests that MoM-LNM may capture subtle phenotypic signatures of metastatic potential beyond conventional pathological assessment, thereby identifying node-negative patients who may require more intensive postoperative surveillance [27].

Nevertheless, these findings should be interpreted with caution. As adjuvant therapy was more common among high-risk patients, the association between MoM-LNM and survival outcomes may be affected by confounding by indication and should not be interpreted as a causal treatment effect. Moreover, the relatively small number of overall survival events compared with recurrence events may limit the precision of multivariable estimates, warranting validation in larger prospective cohorts. Although MoM-LNM showed good discrimination, calibration intercepts deviated from the ideal value of 0 across datasets, which may partly reflect the altered event prevalence resulting from the matched case–control design. Furthermore, although MoM-LNM remained significantly associated with survival after adjustment for LNM status, this finding should be interpreted cautiously because MoM-LNM was originally developed to distinguish pN+ from pN0 patients, and adjustment for LNM status may remove some of the key prognostic information captured by the model. Thus, the adjusted hazard ratios reflect the incremental prognostic value of MoM-LNM beyond nodal status and may conservatively underestimate its overall prognostic potential. Importantly, in the clinically relevant pN0 subgroup, for whom nodal status cannot provide further risk stratification within node-negative disease, MoM-LNM remained strongly and independently associated with RFS, supporting its potential clinical value for identifying high-risk patients with node-negative disease.

The biological relevance of the MoM-LNM model is supported by its association with the tumor immune microenvironment. Bulk RNA-seq and scRNA-seq analyses showed that the high-risk group exhibited a distinct microenvironment characterized by reduced infiltration of inflammatory cells. These findings are consistent with previous studies suggesting that altered anti-tumor immunity may contribute to LUAD progression and metastasis [28,29,30,31]. In addition, enrichment of immune-related pathways and epithelial–mesenchymal transition signatures in the high-risk group provides biological support for the observed association between MoM-LNM risk and LNM. However, given the limited cohort size of scRNA-seq analysis, these findings represent exploratory biological characterization at the cell level rather than definitive patient-level validation. Additionally, the high-risk bulk RNA-seq group was strongly enriched for pN+ cases and advanced-stage disease; thus, the observed transcriptomic differences may reflect underlying clinicopathological confounding rather than causality. Further experimental studies are warranted to clarify the mechanisms linking the MoM-LNM with immune remodeling and metastatic progression.

Several limitations must be noted. First, the 1:2 matched case–control design resulted in an enriched LNM prevalence, which may affect prevalence-dependent metrics (e.g., PPV and NPV) and limit their direct generalizability to routine clinical populations. Second, although survival analysis was adjusted for clinicopathological variables, including adjuvant therapy, residual confounding inherent to retrospective studies cannot be excluded. Third, the pathomics model was developed from postoperative whole-slide images and therefore cannot currently be applied to purely preoperative decision-making. Fourth, tumor segmentation was performed using a standardized semi-automatic workflow with expert review rather than a fully automated approach. Finally, transcriptomic and single-cell RNA sequencing analyses were conducted in relatively small cohorts and should be considered exploratory, requiring further functional validation. In addition, this retrospective study was based on a closed dataset from two centers within a single country. Larger prospective, multicenter studies are needed to validate the robustness and clinical applicability of the proposed model.

## 5. Conclusions

MoM-LNM integrates preoperative CT radiomics and postoperative pathomics to identify LNM and stratify postoperative risk in early-stage LUAD. It identified a high-risk subgroup among pN0 patients and showed an association with distinct immune landscapes, providing biological support for the model. Further prospective and multicenter validation is needed before clinical implementation. If validated, MoM-LNM may improve postoperative risk assessment and help guide surveillance strategies.

## Figures and Tables

**Table 1:** Baseline characteristics of the study patients.

Characteristics	Training Set (*n* = 339)	Validation Set (*n* = 72)	Internal Test Set (*n* = 72)	External Test Set (*n* = 153)
Gender				
Male	125 (36.9%)	35 (48.6%)	40(55.6%)	77 (50.3%)
Female	214 (63.1%)	37 (51.4%)	32 (44.4%)	76 (49.7%)
Age (year)	59 (32–83)	59 (35–75)	60 (24–80)	61 (32–85)
Smoking history	182 (53.7%)	45 (62.5%)	45 (62.5%)	91 (59.5%)
Location				
Left upper lobe	87 (25.7%)	22 (30.6%)	26 (36.1%)	36 (23.5%)
Left lower lobe	42 (12.4%)	15 (20.8%)	6 (8.3%)	16 (10.5%)
Right upper lobe	119 (35.1%)	22 (30.6%)	19 (26.4%)	44 (28.8%)
Right middle lobe	29 (8.6%)	3 (4.2%)	5 (6.9%)	16 (10.5%)
Right lower lobe	62 (18.3%)	10 (13.9%)	16 (22.2%)	41 (26.8%)
Pure-solid nodule				
Yes	150 (44.2%)	53 (73.6%)	35 (48.6%)	35 (22.9%)
Spiculation				
Yes	137 (40.4%)	39 (54.2%)	24 (33.3%)	24 (15.7%)
Lobulation				
Yes	139 (41.0%)	39 (54.2%)	43 (59.7%)	43 (28.1%)
Vacuole sign				
Yes	73 (21.5%)	35 (48.6%)	27 (37.5%)	27 (17.6%)
Stage				
I	234 (69.0%)	39 (54.2%)	48 (66.7%)	102 (66.7%)
II	83 (24.5%)	27 (37.5%)	15 (20.8%)	40 (26.1%)
III	22 (6.5%)	6 (8.3%)	9 (12.5%)	11 (7.2%)
Differentiation				
Well	44 (13.0%)	4 (5.6%)	7 (9.7%)	13 (8.5%)
Moderate	197 (58.1%)	37 (51.4%)	40 (55.6%)	77 (50.3%)
Poor	98 (28.9%)	31 (43.1%)	25 (34.7%)	63 (41.2%)
LNM				
Yes	113 (33.3%)	24 (33.3%)	24 (33.3%)	51 (33.3%)
TLS				
Positive	285 (84.1%)	58 (80.6%)	55 (76.4%)	132 (86.3%)
LVI				
Yes	4 (1.2%)	3 (4.2%)	2 (2.8%)	5 (3.3%)
STAS				
Yes	13 (3.8%)	3 (4.2%)	4 (5.6%)	9 (5.9%)
Pleural invasion				
Yes	9 (2.7%)	3 (4.2%)	2 (2.8%)	7 (4.6%)
Adjuvant therapy				
Yes	85 (25.1%)	26 (36.1%)	16 (22.2%)	35 (22.9%)

Note: Values are given as numbers (percentage%) or mean ± standard deviation. Abbreviations: LNM, lymph node metastasis; TLS, tertiary lymphoid structures; LVI, lymphovascular invasion; STAS, spread through air spaces.

**Table 2:** Univariable and multivariable Cox regression analysis for overall survival and recurrence-free survival.

Variable	OS Univariable HR (95% CI), *p* Value	OS Multivariable HR (95% CI), *p* Value	RFS Univariable HR (95% CI), *p* Value	RFS Multivariable HR (95% CI), *p* Value
Diameter (per 1 mm)	1.052 (0.994–1.112), *p* = 0.078	1.000 (0.938–1.066), *p* = 0.997	1.060 (1.030–1.091), *p* < 0.001	1.007 (0.974–1.041), *p* = 0.672
Differentiation (Poorly vs. Well/Moderately)	2.606 (1.376–4.938), *p* = 0.003	1.276 (0.611–2.665), *p* = 0.516	3.491 (2.498–4.878), *p* < 0.001	(Stratified) *
Lymph node metastasis (Yes vs. No)	4.723 (2.444–9.125), *p* < 0.001	2.331 (0.968–5.616), *p* = 0.059	3.824 (2.696–5.425), *p* < 0.001	(Stratified) *
Lymphovascular invasion (Yes vs. No)	2.181 (0.299–15.941), *p* = 0.442	1.199 (0.156–9.216), *p* = 0.862	3.893 (1.710–8.863), *p* = 0.001	2.648 (1.097–6.395), *p* = 0.030
Spread through air spaces (Yes vs. No)	1.793 (0.242–13.270), *p* = 0.567	0.605 (0.073–5.008), *p* = 0.641	2.392 (1.105–5.176), *p* = 0.027	1.211 (0.525–2.791), *p* = 0.653
Pleural invasion (Yes vs. No)	1.389 (0.190–10.147), *p* = 0.746	1.021 (0.123–8.465), *p* = 0.984	2.851 (1.325–6.130), *p* = 0.007	0.857 (0.370–1.985), *p* = 0.719
Adjuvant therapy (Yes vs. No)	0.615 (0.271–1.399), *p* = 0.247	0.345 (0.147–0.807), *p* = 0.014	2.113 (1.510–2.957), *p* < 0.001	1.457 (1.009–2.103), *p* = 0.045
MoM-LNM risk group (High-risk vs. Low-risk)	5.495 (2.591–11.28), *p* < 0.001	5.128 (1.859–14.286), *p* = 0.002	4.950 (3.448–7.143), *p* < 0.001	(Time-dependent) #
At 12 months (High-risk vs. Low-risk)	-	-	-	3.234 (1.669–6.267), *p* < 0.001
At 24 months (High-risk vs. Low-risk)	-	-	-	2.510 (1.536–4.100), *p* < 0.001
At 36 months (High-risk vs. Low-risk)	-	-	-	2.156 (1.195–3.887), *p* = 0.011

Note: The proportional hazards assumption was violated for the MoM-LNM risk group, lymph node metastasis, and poor differentiation (global test of Schoenfeld residuals, *p* = 1.4 × 10^−8^). Consequently, an extended Cox model with time-dependent covariates was employed for RFS: * lymph node metastasis and poor differentiation were incorporated as stratification factors (no HR estimated for stratification variables), while the # MoM-LNM risk group was modeled as a time-dependent covariate using the counting process formulation. Time-varying hazard ratios for the MoM-LNM risk group are presented at 12, 24, and 36 months. Abbreviations: OS, overall survival; RFS, recurrence-free survival; HR, hazard ratio; CI, confidence interval.

## Data Availability

The datasets generated during the current study are not publicly available due to our institutional regulation, but are available from the corresponding author on reasonable request.

## Supplementary Materials

The following supporting information can be downloaded at: https://www.mdpi.com/article/10.3390/cancers18162720/s1, Figure S1: Complete Pipeline of the Model. * All feature extraction, normalization, and model training steps were performed exclusively within the training fold of each cross-validation iteration. CT, computed tomography; MIL, multiple-instance learning; WSI, whole-slide image; ICC, intraclass correlation coefficient; LASSO, Least Absolute Shrinkage and Selection Operator; AUC, area under curve; DCA, decision curve analysis Figure S2: Feature selection. (A,B) The results of LASSO regression for radiomics feature selection. (C,D) The results of the autoencoder for pathomics feature selection. LASSO, Least Absolute Shrinkage and Selection Operator. Figure S3: DCA curves before prevalence adjustment. (A–D) DCA of the three models in the training set (A), validation set (B), internal test set (C), and external test set (D). Figure S4: Calibration curves of the multimodal model. Calibration curves for training. (A) Calibration plots for the training, validation, internal test, and external test sets under the original 1:2 case-control sampling framework. (B) Calibration curves recalibrated to reflect the true LNM prevalence (10.2% for internal sets; 8.8% for the external set). Figure S5: Kaplan–Meier survival curves in the independent external test set and the propensity score-matched cohort. Overall survival (A) and recurrence-free survival (B) of the high-risk and low-risk groups in the independent external validation cohort. Overall survival (C) and recurrence-free survival (D) of the high-risk and low-risk groups in the propensity score-matched cohort. OS, overall survival; RFS, recurrence-free survival. Figure S6: Cell distributions across all patients. UMAP, uniform manifold approximation and projection. Figure S7: The major enriched pathways of malignant cells. Figure S8: Intercellular communication networks of the high- and low-risk groups. Table S1: Clinical characteristics of lung adenocarcinoma patients with single cell sequencing. Table S2: Clinical characteristics of lung adenocarcinoma patients with RNA sequencing. Table S3: The performance of models in identifying lymph node metastasis. Table S4: Confusion matrices of the multimodal radiology–pathology model for lymph node metastasis prediction. Table S5: Delong’s test in different models. Table S6: Calibration performance of the multimodal radiology–pathology model across different datasets. Table S7: Distribution of adjuvant therapy between MoM-LNM risk groups. Table S8: Comparison of prognostic performance between conventional clinicopathological models with and without MoM-LNM.

## Abbreviations

The following abbreviations are used in this manuscript:

CT	computed tomography
LNM	lymph node metastasis
LUAD	lung adenocarcinoma
RFS	recurrence-free survival
IAC	invasive adenocarcinoma
WSI	whole-slide image
HE	hematoxylin and eosin
TNM	tumor-node-metastasis
Bulk RNA-seq	bulk RNA sequencing
ScRNA-seq	single-cell RNA sequencing
TLS	tertiary lymphoid structures
LVI	lymphovascular invasion
STAS	spread through air spaces
OS	overall survival
ICCs	intraclass correlation coefficients
LASSO	Least Absolute Shrinkage and Selection Operator
MLP	multilayer perceptron
AUC	area under curve
ROC	receiver operating characteristic curve analysis
PPV	positive predictive value
NPV	negative predictive value
DCA	decision curve analysis
SHAP	SHapley Additive exPlanations
UMAP	uniform manifold approximation and projection
DEGs	differentially expressed genes
GO	Gene Ontology
KEGG	Kyoto Encyclopedia of Genes and Genomes
GSEA	Gene Set Enrichment Analysis
ssGSEA	single-sample GSEA
SD	standard deviation
OR	odds ratio
PSM	propensity score matching
SMD	standardized mean difference
pN+	pathologically confirmed nodal metastasis
pN0	pathologically confirmed no nodal metastasis

## Appendix A

### Appendix A.1. Radiomics Model

The radiomics signature was implemented using a multilayer perceptron (MLP) consisting of two hidden layers (64 and 32 neurons with ReLU activation) followed by a Sigmoid output layer. Grid search identified the optimal hyperparameters as a batch size of 25, learning rate of 0.05, and dropout rate of 0.25.

### Appendix A.2. Pathomics Model

The pathomics signature adopted the same MLP architecture. The optimal hyperparameters were a batch size of 35, learning rate of 0.01, and dropout rate of 0.75.

### Appendix A.3. Multimodal Model

The multimodal classifier also employed the same MLP architecture. The final model was trained using the Adam optimizer (learning rate = 1 × 10^−3^), a batch size of 32, binary cross-entropy loss, and dropout probabilities of 0.2 and 0.4 after the first and second hidden layers, respectively. The model achieving the highest validation AUC during 100 epochs was selected.

## Appendix B

Fresh tumor specimens were washed with cold DPBS and minced into ~1 mm^3^ pieces. The tissues were enzymatically digested at 37 °C for 30–45 min in DPBS containing 1–2 mg mL^−1^ collagenase IV and 0.1 mg mL^−1^ DNase I with gentle agitation. The digested samples were gently triturated and passed through 70 μm and 40 μm cell strainers to remove debris. Cells were centrifuged at 300× *g* for 5 min and resuspended in DPBS containing 0.04% BSA. Red blood cells were lysed using ACK lysis buffer when necessary. Cell viability was assessed by trypan blue staining, and samples with viability >85% were used for downstream processing. If required, dead cells were removed using a commercial dead cell removal kit according to the manufacturer’s instructions. Single-cell suspensions were loaded into Chromium microfluidic chips using the Next GEM Single Cell 3′ v3.1 chemistry and barcoded on a 10× Chromium Controller (10× Genomics, Pleasanton, CA, USA). Reverse transcription and library construction were performed using the Chromium Next GEM Single Cell 3′ Kit v3.1 (10x Genomics, Pleasanton, CA, USA) following the manufacturer’s protocol. Sequencing was conducted on an Illumina NovaSeq 6000 platform (Illumina, San Diego, CA, USA) according to standard procedures.

## Appendix C

High-throughput sequencing was performed by Seqhealth Technology Co., Ltd. (Wuhan, China). RNA Extraction, Library Preparation, and Sequencing.

Total RNA was isolated from the specified biological materials using TRIzol Reagent (Thermo Fisher Scientific, Waltham, MA, USA, cat. no. 15596026) following the manufacturer’s recommended protocol. Subsequent to RNA extraction, genomic DNA was removed by treating the samples with DNase I (NEB, Ipswich, MA, USA, cat. no. M0303L) to eliminate potential DNA contamination. The purity of the extracted RNA was assessed by measuring the A260/A280 ratio using a NanoDrop One spectrophotometer (Thermo Fisher Scientific, Waltham, MA, USA). The integrity of the RNA was verified using the LabChip GX Touch system (Revvity, Waltham, MA, USA). The concentration of the qualified RNA was then determined using a Qubit 3.0 fluorometer with the QubitTM RNA Broad Range Assay kit (Thermo Fisher Scientific, cat. no. Q10210).

For library preparation, the total RNA was used as input for RNA sequencing library preparation utilizing the KC Digital mRNA Library Prep Kit (Seqhealth Tech. Co., Ltd., Wuhan, China), according to the manufacturer’s instructions. The kit uses a 12-base unique molecular identifier (UMI) to label individual cDNA molecules, thereby reducing PCR duplication bias and sequencing errors. The library preparation included the PCR products with insert sizes of 200–500 bp were enriched. The enriched libraries were quantified and sequenced on a NovaSeq X Plus platform (Illumina) using the PE150 sequencing mode to generate paired-end reads.
