# Supplementary material for: Integrated Radiologic, Pathologic, and Transcriptomic Analysis of Lymph Node Metastasis Potential in Small (≤3 cm) Lung Adenocarcinoma
Source: Cancers (Basel). 2026 Aug 21;18(16):2720. doi: 10.3390/cancers18162720 (PMC13511339; doi:10.3390/cancers18162720)
Supplement: Supplementary file 1 [file cancers-18-02720-s001.zip › cancers-4438677-supplementary.pdf]

Supplementary Material

Supplementary Table S1. Clinical characteristics of lung adenocarcinoma patients with single-cell sequencing.

Characteristics	Patient 1	Patient 2	Patient 3	Patient 4	Patient 5	Patient 6
Age	60	58	38	65	71	62
Sex	Female	Male	Male	Female	Female	Female
Smoking history	No	Yes	Yes	No	No	No
Tumor size (mm)	20	19	26	29	11	23
pT	pT2a	pT1b	pT1c	pT1c	pT1b	pT1c
Stage	IB	IA2	IA3	IA3	IA1	IA3
Tumor differentiation	Moderately differentiated	Poorly differentiated	Moderately differentiated	Moderately differentiated	Moderately differentiated	Moderately differentiated
Predominant pattern	Papillary pattern	Solid pattern	Acinar pattern	Acinar pattern	Acinar pattern	Lepidic pattern
Solid pattern (%)	0	60	0	0	0	0
Micropapillary (%)	0	0	0	0	0	0
STAS	Yes	Yes	No	No	No	No
LVI	Yes	No	No	No	No	No
Pleural invasion	Yes	No	No	No	No	No
LNM	No	No	No	No	No	No
EGFR/KRAS mutation	No	No	NA	No	NA	NA
Number of retained cells	16254	12751	10530	12597	11480	10852
Mutimodal model	High-risk	High-risk	High-risk	Low-risk	Low-risk	Low-risk

Supplementary Table S2. Clinical characteristics of lung adenocarcinoma patients with RNA sequencing.

Variables	Multimodal model (High-risk) (n=8)	Multimodal model (Low-risk) (n=8)	*p*
Age	62.2 ± 7.4	58.6 ± 8.9	0.390
Gender			1.000
Male	3 (37.5%)	2 (25.0%)	
Female	5 (62.5%)	6 (75.0%)	
Tumor location			0.748
Left upper lobe	2 (25.0%)	1 (12.5%)	
Left lower lobe	2 (25.0%)	0 (0.0%)	
Right upper lobe	1 (12.5%)	2 (25.0%)	
Right middle lobe	2 (25.0%)	3 (37.5%)	
Right lower lobe	1 (12.5%)	2 (25.0%)	
Stage			0.326
I	1 (12.5%)	4 (50.0%)	
II	4 (50.0%)	3 (37.5%)	
III	3 (37.5%)	1 (12.5%)	
LNM			0.041

Yes	7 (87.5%)	2 (25.0%)	
No	1 (12.5%)	6 (75.0%)	
Tumor differentiation			0.308
Well differentiated	0 (0.00%)	2 (25.0%)	
Moderately differentiated	5 (62.5%)	5 (62.5%)	
Poor differentiated	3 (37.5%)	1 (12.5%)	

Note: Values are given as numbers (%) or mean ± standard deviation. LNM, lymph node metastasis.

Supplementary Table S3. The performance of models in identifying lymph node metastasis.

Cohort	Model	AUC (95%CI)	Accuracy	Sensitivity	Specificity	PPV[a]	NPV[a]	PPV[b]	NPV[b]
Training set	Radiomics	0.808 (0.753-0.856)	0.797 (0.750-0.836)	0.694 (0.598-0.770)	0.849 (0.797-0.890)	0.700 (0.606-0.774)	0.845 (0.789-0.890)	0.343 (0.251-0.443)	0.961 (0.946-0.971)
	Pathomics	0.897 (0.859-0.930)	0.845 (0.804-0.881)	0.883 (0.813-0.932)	0.826 (0.772-0.872)	0.721 (0.635-0.791)	0.933 (0.890-0.962)	0.366 (0.288-0.453)	0.984 (0.973-0.991)
	MoM-LNM	0.933 (0.899-0.961)	0.909 (0.874-0.935)	0.929 (0.866-0.964)	0.898 (0.853-0.931)	0.820 (0.743-0.878)	0.961 (0.927-0.980)	0.513 (0.401-0.613)	0.991 (0.982-0.996)
Validation set	Radiomics	0.891 (0.821-0.950)	0.807 (0.697-0.883)	1.000 (0.858-1.000)	0.714 (0.568-0.817)	0.628 (0.468-0.771)	1.000 (0.900-1.000)	0.284 (0.184-0.383)	1.000 (0.972-1.000)
	Pathomics	0.921 (0.861-0.973)	0.867 (0.760-0.925)	1.000 (0.858-1.000)	0.804 (0.652-0.889)	0.711 (0.538-0.834)	1.000 (0.908-1.000)	0.367 (0.219-0.506)	1.000 (0.976-1.000)
	MoM-LNM	0.944 (0.894-0.986)	0.903 (0.813-0.953)	0.958 (0.789-0.993)	0.875 (0.755-0.941)	0.793 (0.608-0.902)	0.977 (0.882-0.996)	0.467 (0.268-0.657)	0.995 (0.969-0.999)
Internal test set	Radiomics	0.722 (0.567-0.855)	0.797 (0.697-0.883)	0.667 (0.467-0.820)	0.867 (0.753-0.941)	0.727 (0.513-0.876)	0.830 (0.715-0.916)	0.363 (0.177-0.612)	0.958 (0.926-0.979)
	Pathomics	0.807 (0.698-0.896)	0.812 (0.715-0.890)	0.958 (0.789-0.993)	0.733 (0.611-0.853)	0.657 (0.486-0.796)	0.971 (0.862-0.995)	0.290 (0.187-0.434)	0.994 (0.962-0.999)
	MoM-LNM	0.879 (0.772-0.959)	0.875 (0.779-0.933)	0.792 (0.595-0.908)	0.917 (0.804-0.968)	0.826 (0.630-0.930)	0.898 (0.787-0.954)	0.503 (0.256-0.763)	0.975 (0.946-0.989)
Ext	Ra	0.730	0.662	0.755	0.618	0.487	0.840	0.160	0.963

ernal test set	diomics	(0.630-0.821)	(0.579-0.732)	(0.608-0.847)	(0.521-0.707)	(0.382-0.605)	(0.731-0.897)	(0.109-0.218)	(0.932-0.980)
	Pathomics	0.776 (0.691-0.855)	0.709 (0.635-0.779)	0.837 (0.713-0.914)	0.647 (0.548-0.735)	0.532 (0.433-0.651)	0.892 (0.801-0.945)	0.186 (0.132-0.250)	0.976 (0.952-0.989)
	MoM-LNM	0.893 (0.831-0.947)	0.856 (0.791-0.904)	0.882 (0.765-0.945)	0.843 (0.762-0.902)	0.738 (0.618-0.831)	0.935 (0.866-0.970)	0.350 (0.237-0.482)	0.986 (0.971-0.994)

Note: Values are given as numbers (percentage%) or mean ± standard deviation. [a.]Calculated based on the study cohort (case-to-control ratio = 1:2; prevalence ≈ 33.3%). [b]Adjusted to the estimated real-world prevalence of lymph node metastasis (10.2% for training, validation, and internal test sets; 8.8% for external test set) using Bayes' theorem. Abbreviations: AUC, area under the curve; CI, confidence interval; PPV, positive predictive value; NPV, negative predictive value.

Supplementary Table S4. Confusion matrices of the multimodal radiology–pathology model for lymph node metastasis prediction.

Dataset		Actual Positive	Actual Negative
Training set	Predicted Positive	105	23
	Predicted Negative	8	203
Validation set	Predicted Positive	23	6
	Predicted Negative	1	42
Internal test set	Predicted Positive	19	4
	Predicted Negative	5	44
External test set	Predicted Positive	45	16
	Predicted Negative	6	86

Supplementary Table S5. DeLong's test in different models.

	Z	P
Training dataset		
Pathomics vs Radiomics	1.96	0.050
Pathomics vs Multimodal	-0.910	0.363
Radiomics vs Multimodal	-3.136	0.002
Validation dataset		
Pathomics vs Radiomics	0.322	0.748
Pathomics vs Multimodal	-0.337	0.736
Radiomics vs Multimodal	-0.639	0.523
Internal test dataset		
Pathomics vs Radiomics	0.983	0.326
Pathomics vs Multimodal	-0.972	0.331
Radiomics vs Multimodal	-2.269	0.023
External test dataset		
Pathomics vs Radiomics	0.690	0.490
Pathomics vs Multimodal	-2.119	0.034
Radiomics vs Multimodal	-2.701	0.006

Supplementary Table S6. Calibration performance of the multimodal radiology–pathology model across different datasets.

	Original calculation (Study cohort: 1:2 ratio; Prevalence=33.3%)			Benchmark adjusted to real-world prevalence (LNM: 10.2%% train/val/test; 8.8% external)		
Dataset	Brier Score	Calibration Intercept (95% CI)	Calibration Slope (95% CI)	Brier Score	Calibration Intercept (95% CI)	Calibration Slope (95% CI)
Training set	0.137	1.518 (1.036-1.999)	1.000 (0.781-1.219)	0.129	1.371 (0.911-1.831)	1.000 (0.781-1.219)
Validation set	0.128	1.754 (0.737-2.772)	1.570 (0.746-2.394)	0.117	1.446 (0.532-2.359)	1.570 (0.746-2.394)
Internal test set	0.167	0.881 (0.052-1.710)	0.892 (0.384-1.401)	0.161	0.796 (-0.000-1.591)	0.892 (0.384-1.401)
External test set	0.167	1.222 (0.578-1.865)	0.751 (0.499-1.002)	0.166	1.215 (0.573-1.858)	0.751 (0.499-1.002)

Note: Calibration intercept values close to 0 and calibration slopes close to 1 indicate good calibration. Training cohort calibration metrics were calculated using out-of-fold predictions.

Supplementary Table S7. Distribution of adjuvant therapy between MoM-LNM risk groups.

Variable	High-risk group (n=271)	Low-risk group (n=365)	Standardized mean difference
Adjuvant therapy			0.531
Yes	105 (38.7%)	57 (15.6%)	
No	166 (61.3%)	308 (84.4%)	

Supplementary Table S8. Comparison of prognostic performance between conventional clinicopathological models with and without MoM-LNM.

Metric	Overall survival		Recurrence-free survival	
	Model 1	Model 2	Model 1	Model 2
C-index (95% CI)	0.702 (0.606-0.797)	0.754 (0.658-0.849)	0.673 (0.624-0.722)	0.694 (0.645-0.743)
ΔC-index (95% CI)	NA	0.052 (-0.005-0.105)	NA	0.021 (0.005-0.060)
P value for ΔC-index	NA	0.188	NA	0.006
Log-likelihood	-221.259	-218.186	-840.328	-829.000
AIC	454.518	450.373	1692.655	1672.000
BIC	481.250	481.559	1719.386	1703.187
Likelihood ratio χ^2	NA	6.146	NA	22.655
LR test P value	NA	0.013	NA	<0.001

Note: Model 1 was the conventional clinicopathological model including tumor diameter, differentiation, lymph node metastasis, lymphovascular invasion, spread through air spaces, and pleural invasion. Model 2 additionally incorporated MoM-LNM. ΔC-index represents the difference in C-index between Model 2 and

Model 1.

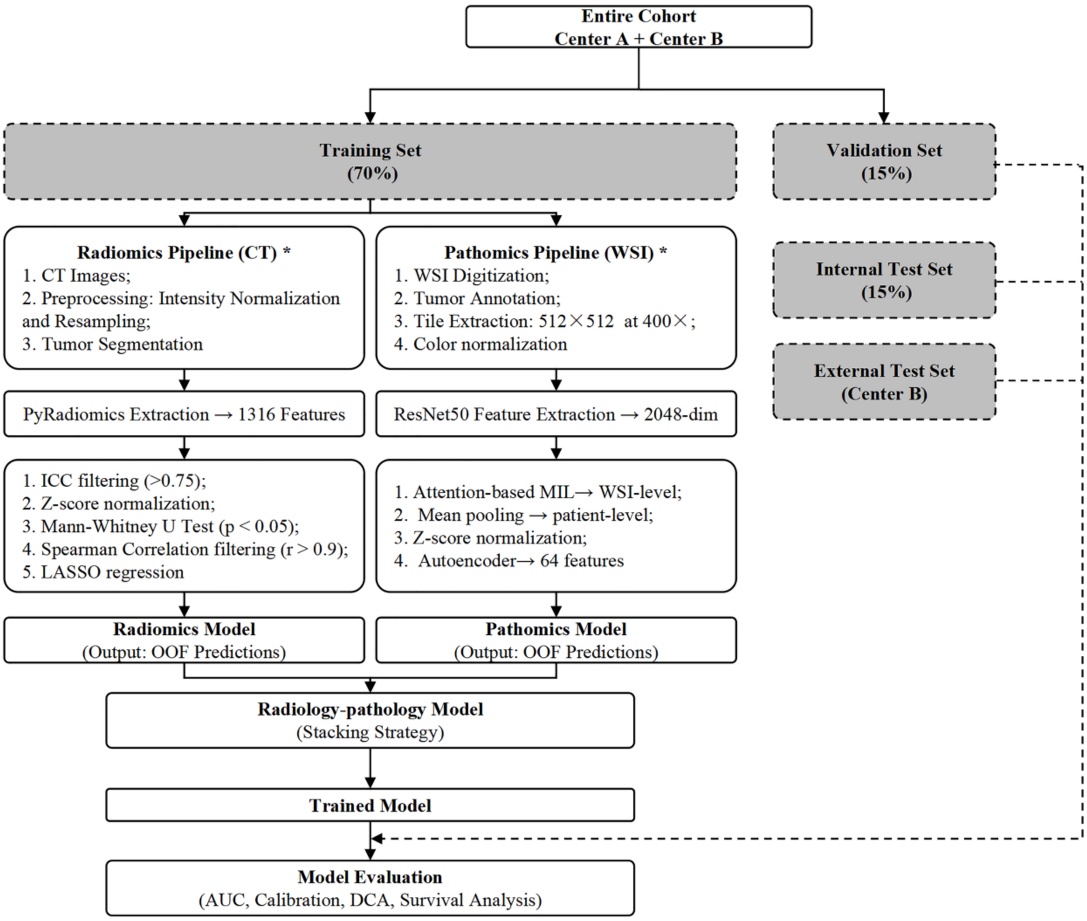

Supplementary Figure S1. Complete Pipeline of the Model. * All feature extraction, normalization, and model training steps were performed exclusively within the training fold of each cross-validation iteration. CT, computed tomography; MIL, multiple-instance learning; WSI, whole-slide image; ICC, intraclass correlation coefficient; LASSO, Least Absolute Shrinkage and Selection Operator; AUC, area under curve; DCA, decision curve analysis.

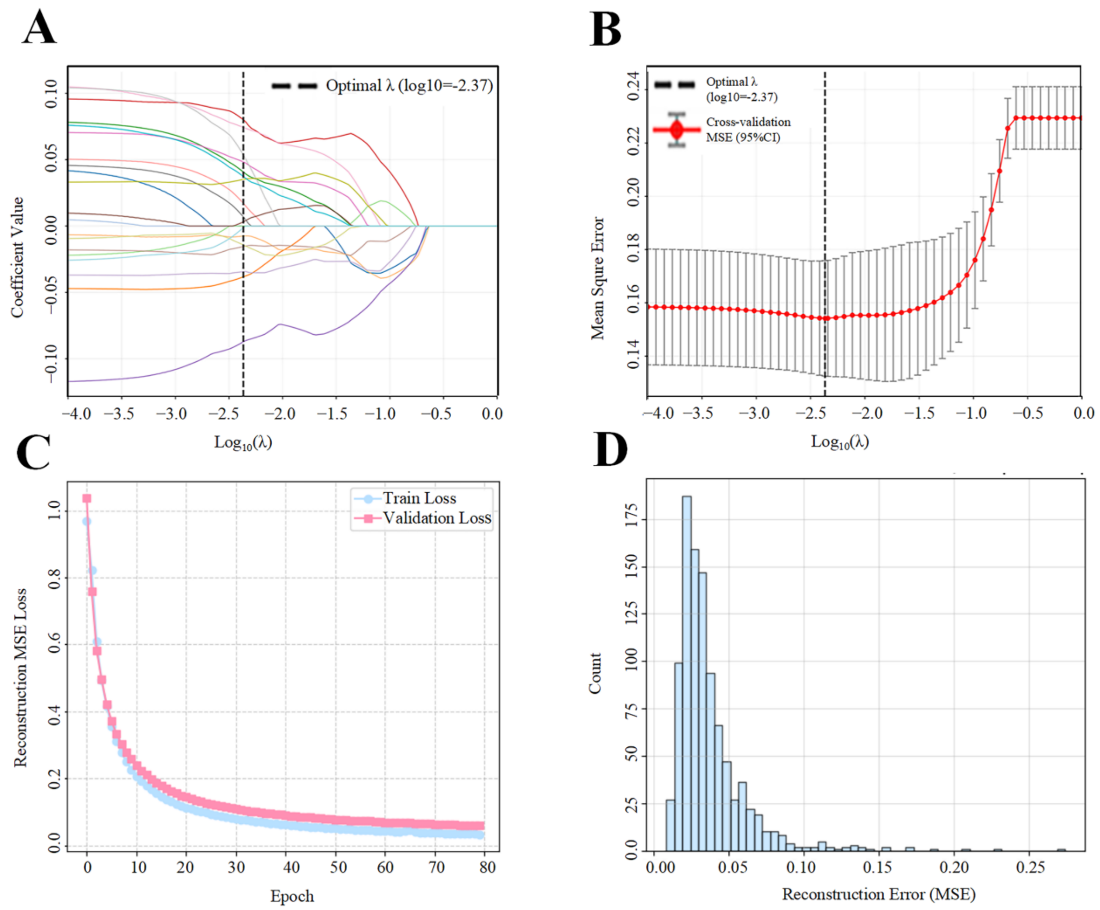

Supplementary Figure S2. Feature selection. (A,B) The results of LASSO regression for radiomics feature selection. (C,D) The results of the autoencoder for pathomics feature selection. LASSO, Least Absolute Shrinkage and Selection Operator.

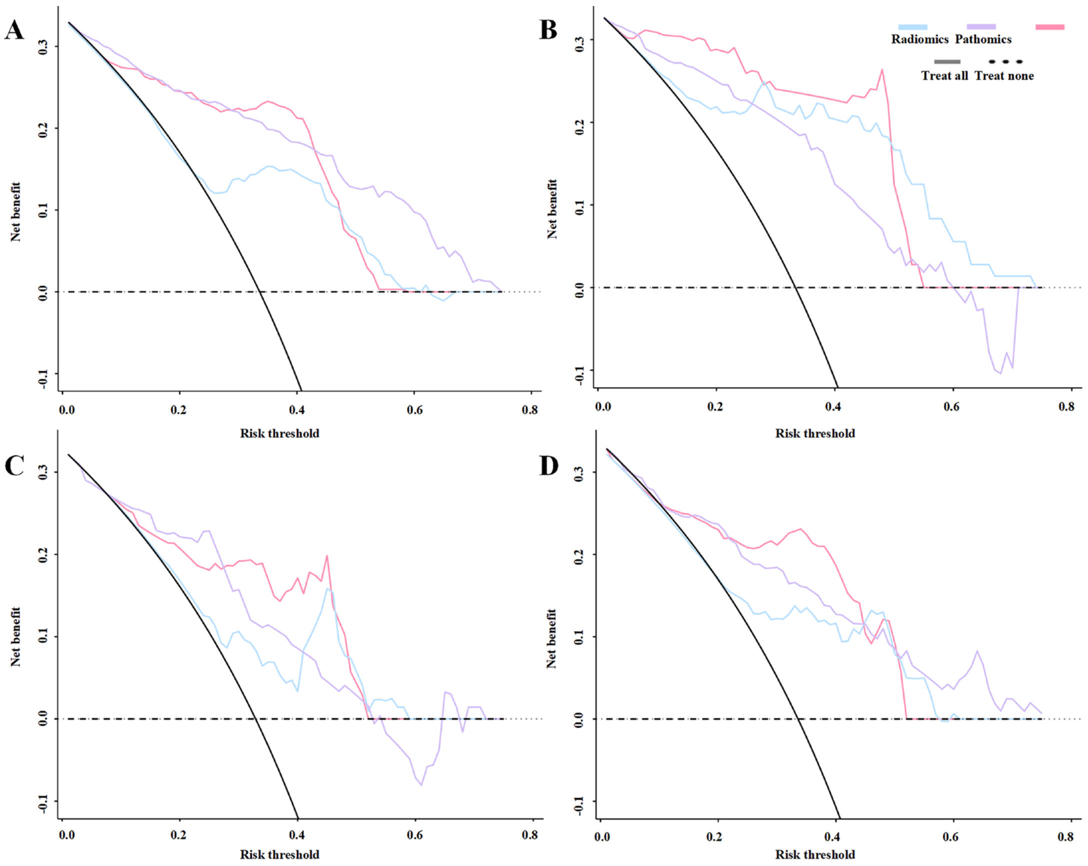

Supplementary Figure S3. DCA curves before prevalence adjustment. (A–D) DCA of the three models in the training set (A), validation set (B), internal test set (C), and external test set (D).

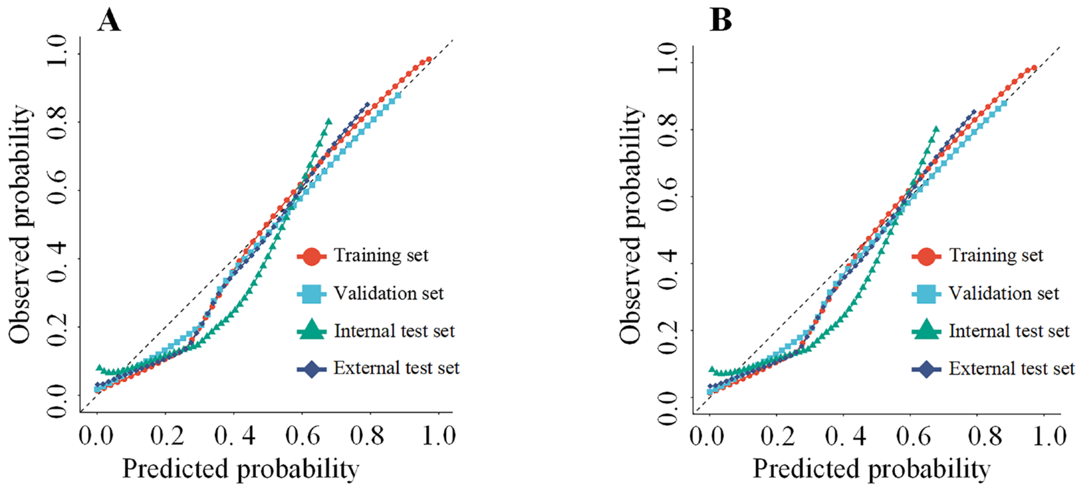

Supplementary Figure S4. Calibration curves of the multimodal model. Calibration curves for training. (A) Calibration plots for the training, validation, internal test, and external test sets under the original 1:2 case-control sampling framework. (B) Calibration curves recalibrated to reflect the true LNM prevalence (10.2% for internal sets; 8.8% for the external set).

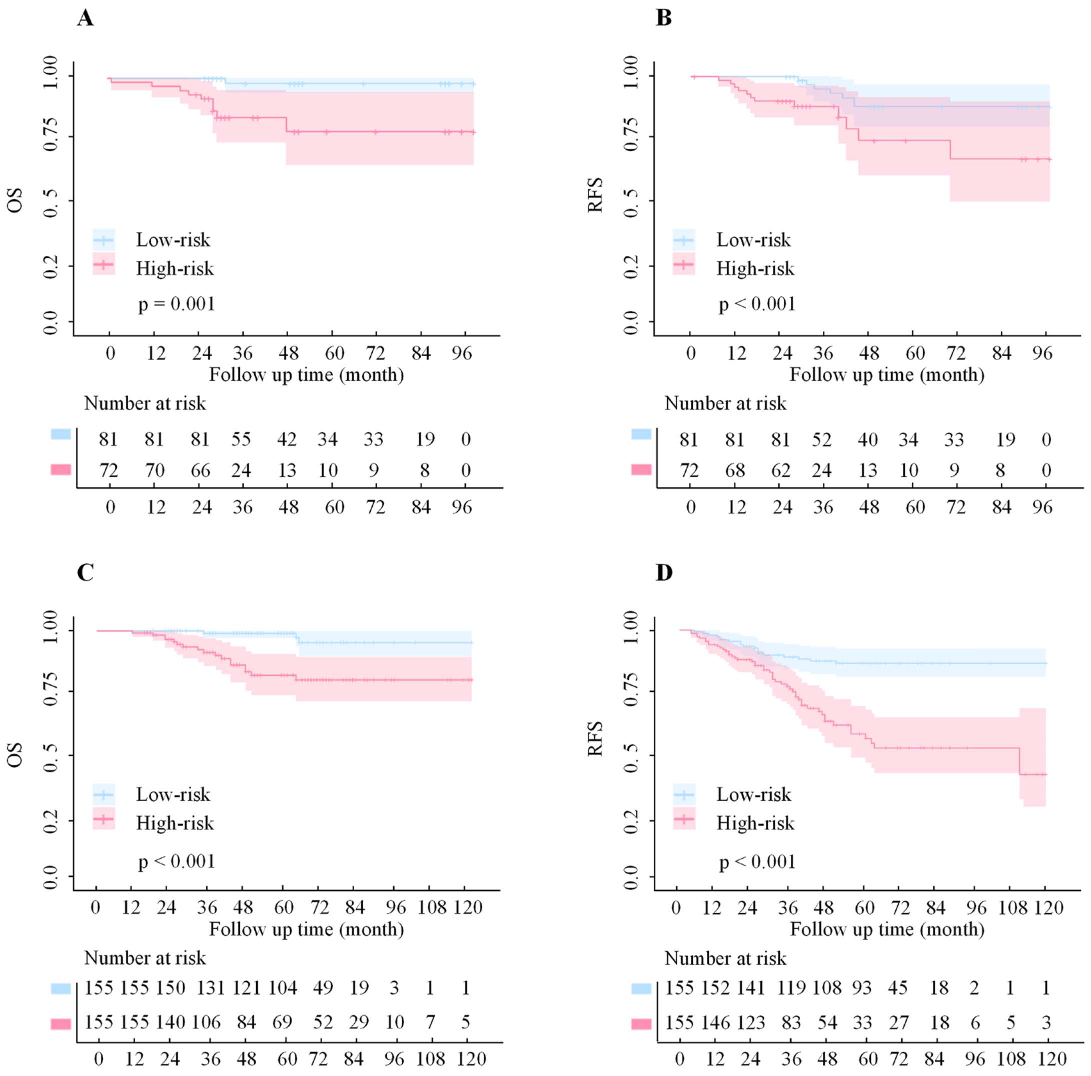

Supplementary Figure S5. Kaplan–Meier survival curves in the independent external test set and the propensity score-matched cohort. Overall survival (A) and recurrence-free survival (B) of the high-risk and low-risk groups in the independent external validation cohort. Overall survival (C) and recurrence-free survival (D) of the high-risk and low-risk groups in the propensity score-matched cohort. OS, overall survival; RFS, recurrence-free survival.

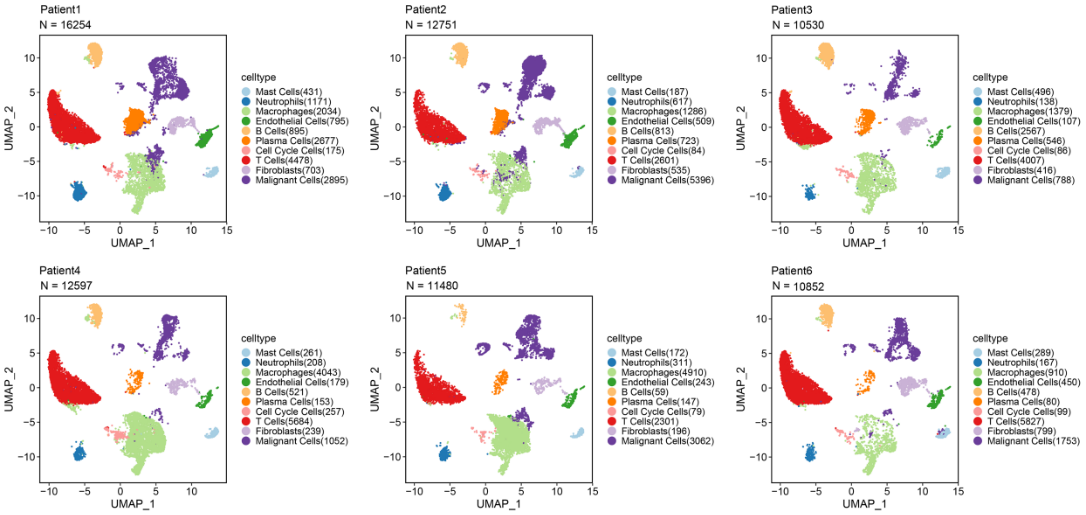

Supplementary Figure S6. Cell distributions across all patients. UMAP, uniform manifold approximation and projection.

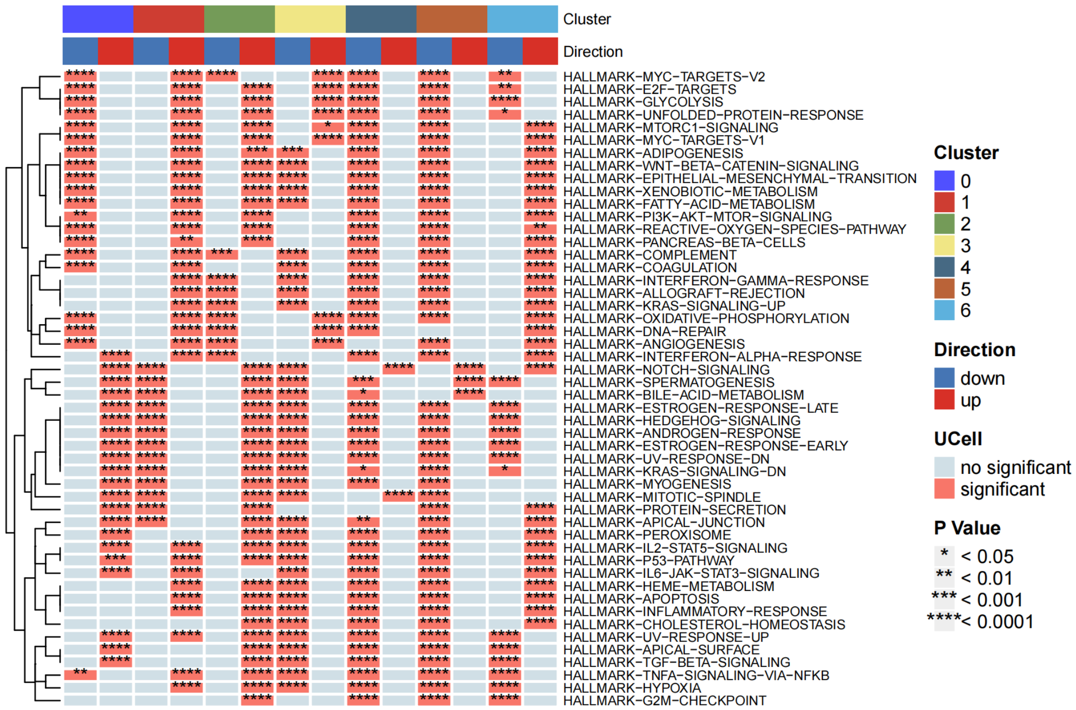

Supplementary Figure S7. The major enriched pathways of malignant cells.

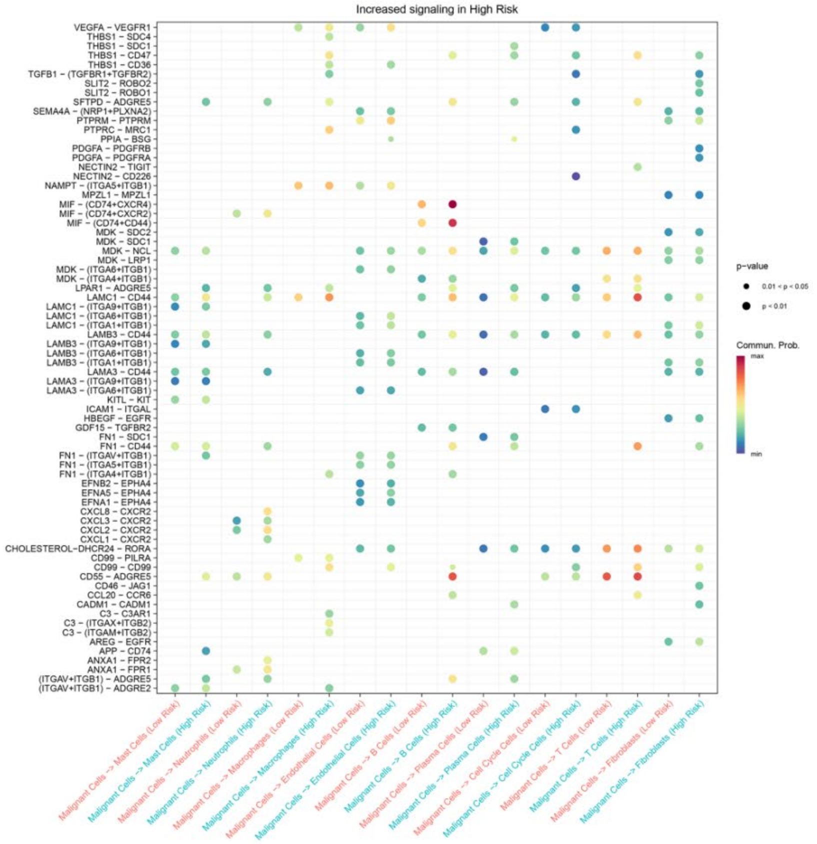

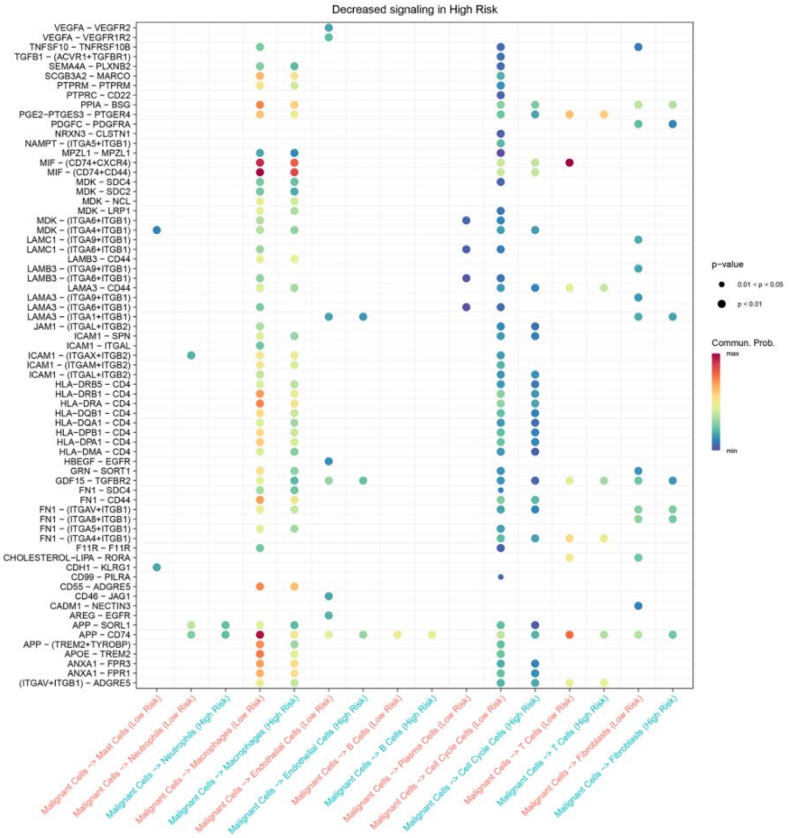

Supplementary Figure S8. Intercellular communication networks of the high- and low-risk groups.
